# Gravitational Force—Induced 3D Chromosomal Conformational Changes Are Associated with Rapid Transcriptional Response in Human T Cells

**DOI:** 10.3390/ijms22179426

**Published:** 2021-08-30

**Authors:** Christian Vahlensieck, Cora Sandra Thiel, Ye Zhang, Andreas Huge, Oliver Ullrich

**Affiliations:** 1 Institute of Anatomy, Faculty of Medicine, University of Zurich, Winterthurerstrasse 190, 8057 Zurich, Switzerland; christian.vahlensieck@uzh.ch; 2 Innovation Cluster Space and Aviation (UZH Space Hub), Air Force Center, University of Zurich, Überlandstrasse 271, 8600 Dübendorf, Switzerland; 3 Space Life Sciences Laboratory (SLSL), Kennedy Space Center (KSC), 505 Odyssey Way, Exploration Park, FL 32953, USA; 4 ISS Utilization and Life Sciences Division, National Aeronautics and Space Administration (NASA), Kennedy Space Center, FL 32899, USA; ye.zhang-1@nasa.gov; 5 Core Facility Genomic, Medical Faculty of Muenster, University of Muenster, Albert-Schweitzer-Campus 1, D3, Domagstrasse 3, 48149 Muenster, Germany; a.huge@uni-muenster.de; 6 Space Biotechnology, Department of Machine Design, Engineering Design and Product Development, Institute of Mechanical Engineering, Otto-von-Guericke-University Magdeburg, Universitätsplatz 2, 39106 Magdeburg, Germany; 7 Space Medicine, Ernst-Abbe-Hochschule (EAH) Jena, Department of Industrial Engineering, Carl-Zeiss-Promenade 2, 07745 Jena, Germany; 8 Zurich Center for Integrative Human Physiology (ZIHP), University of Zurich, Winterthurerstrasse 190, 8057 Zurich, Switzerland

**Keywords:** immune cells, gravity-sensing, mechanosensing, microgravity, spaceflight, altered gravity, gene expression

## Abstract

The mechanisms underlying gravity perception in mammalian cells are unknown. We have recently discovered that the transcriptome of cells in the immune system, which is the most affected system during a spaceflight, responds rapidly and broadly to altered gravity. To pinpoint potential underlying mechanisms, we compared gene expression and three-dimensional (3D) chromosomal conformational changes in human Jurkat T cells during the short-term gravitational changes in parabolic flight and suborbital ballistic rocket flight experiments. We found that differential gene expression in gravity-responsive chromosomal regions, but not differentially regulated single genes, are highly conserved between different real altered gravity comparisons. These coupled gene expression effects in chromosomal regions could be explained by underlying chromatin structures. Based on a high-throughput chromatin conformation capture (Hi-C) analysis in altered gravity, we found that small chromosomes (chr16–22, with the exception of chr18) showed increased intra- and interchromosomal interactions in altered gravity, whereby large chromosomes showed decreased interactions. Finally, we detected a nonrandom overlap between Hi-C-identified chromosomal interacting regions and gravity-responsive chromosomal regions (GRCRs). We therefore demonstrate the first evidence that gravitational force-induced 3D chromosomal conformational changes are associated with rapid transcriptional response in human T cells. We propose a general model of cellular sensitivity to gravitational forces, where gravitational forces acting on the cellular membrane are rapidly and mechanically transduced through the cytoskeleton into the nucleus, moving chromosome territories to new conformation states and their genes into more expressive or repressive environments, finally resulting in region-specific differential gene expression.

## 1. Introduction

The gravitational force has been constant throughout the four billion years of Earth’s evolutionary history [1] and probably played a crucial role in the evolutionary development and adaptation of all terrestrial life [2,3]. The gravitational force substantially affects plants [4,5], protozoa [6], and mammalian cells [7], where microgravity has been demonstrated to have profound effects at the cellular and molecular levels [8]. Whereas the perception of gravity in plants and protozoa is widely investigated [6,7] with gravity-sensory ion channels, starch-statolith (reviewed in [9]), and cascades of second messengers identified in some cases [6], the mechanisms of gravity perception in mammalian cells are unknown. One robust hypothesis proposed the cytoskeleton as an organized and stabilized tension-dependent gravity-sensing architecture [10], where forces are mediated by both intermediate filaments and F-actin [11,12]. It has been also proposed that mechanosensitive ion channels [13], changes in the cell membranes fluidity [14], transcription factors [15], and transcriptional and translational regulations via noncoding RNA mechanisms [16] are involved in cellular sensitivity to gravity.

We previously discovered that cells of the immune system, one of the most affected systems during spaceflight [17,18], respond rapidly to altered gravity regarding signal transduction cascades [19,20,21,22], cell cycle control proteins [23], epigenetic modifications [22], adhesion molecule expression [24,25,26], and the oxidative burst reaction [27,28]. Recently, we detected profound rapid alterations of the transcriptome in human Jurkat T cells and human myelo-monocytic U937 cells under altered gravity conditions. Differential gene expressions (DGEs) in these cell lines were detectable after 20 s of altered gravity during a parabolic flight as well as after 75 s of hypergravity and 300 s of microgravity during a suborbital ballistic rocket flight. These alterations affect a major fraction of transcripts without significant overlaps and functional relations between the patterns of different time points [29,30,31,32,33,34,35]. The findings are independent of ion channel function [31,33,34]. Our data suggested a rapid coupling between the gravitational force and the transcriptome [34] after an initial trigger mechanism of less than 1 s reaction time [30].

Indeed, mechanical force transduction into the chromatin occurs within seconds [36], allowing the nuclear structure to respond directly without biochemical signaling [37,38]. On the other side, we recently found that microgravity induces structural cellular changes with the rapid response and adaptation of the potential gravity-transducing cytoskeleton [39]. However, besides the cytoskeleton, other cellular structures such as the entire cell membrane, the nuclear membrane, and the actin inside the nucleus are most likely involved in altered gravity induced changes. Because small forces in the low piconewton range may still trigger nuclear mechanotransduction [38], it is possible that the small altered gravitational force could be sensed and transduced through the cellular [10,40,41] and nuclear mechanical architectures [42,43,44]. This mechanotransduction induced by altered gravitational force could then alter nuclear plasticity, chromatin organization and accessibility, and subsequently gene expression [36,45,46,47,48,49]. The fact that the nucleus is obviously subjected to permanent forces induced by Earth’s gravity, either directly or indirectly, therefore leading to the fundamental question as to whether the gravitational force on Earth plays a role in stabilizing and maintaining chromatin architecture and accessibility, and therefore, constitutes a homeostatic condition for gene expression.

Assuming that multiple simultaneous inputs are transduced and integrated within the structural complexity of the living cell [10] and that the gravitational force acts everywhere in and around the cell, the question as to how such a non-specific force signal is finally transduced into a highly-specific gene expression response arises. We therefore investigated the hypothesis that the rapid genomic response to altered gravitational forces is encoded in the organization of the chromatin. The model system required for this purpose must therefore enable the investigation of very rapid effects of gravitational changes. The selection of the appropriate model system, especially in gravitational biology, requires very careful considerations and a full understanding of the assumptions associated with the applied model system.

Experiments in the investigated ultrashort time frame by means of two-dimensional (2D) clinostat or random positioning machine (RPM) experiment are technically not possible or only possible to a limited extent, due to the technical time lag phase to reach or end the vector-averaged gravity state. Simulation devices such as the RPM are typically used to study processes on the timescale of hours and are not well suited to analyze very short-term microgravity effects, since they have relatively slow gravity state transition, which takes about 10–15 min for the averaged g vector to reach a microgravity-relevant level [50]. Regarding the 2D clinostat system, our previous work provided clear empirical evidence that the transduction and response times to gravity changes are faster than 1 s in the Jurkat T cell model system [30]. Furthermore, this fast response time of less than 1 s is consistent with measurements of the oxidative burst reaction in NR8383 macrophages on both a parabolic flight experiment [27] and an International Space Station (ISS) experiment [28]. The initially altered gene expression response and the oxidative burst reaction adapted to the new gravity environment within 5 min and 42 s, respectively. Furthermore, our previous studies also revealed that less than 1% of all transcript clusters demonstrated the same response in simulated (2D clinostat) and real (suborbital ballistic rocket) microgravity after a 5 min exposure time, while 38% of all differentially regulated transcript clusters during the hypergravity phase of the suborbital ballistic rocket flight could be reproduced with 9× *g* ground centrifuge experiments [30]. In line with these findings, the oxidative burst reaction in mammalian macrophage adapted only in real microgravity, as we have demonstrated in two independent flight missions: on the ISS [28] and on a parabolic flight [27], but not in clinostat simulations [51,52]. This is not surprising, since comparability with real microgravity requires that the direction changes are faster than the response time of the system to gravity field [53], which means that for a rotation speed of 60 rpm, the biological effect of gravitational forces is canceled if the initial trigger mechanism requires at least one second, according to the clinostat theory. Therefore, weightlessness simulators are very difficult to use both from a physical (due to the necessary trigger time of >1 s) and operational point of view (time delays due to the need to ramp up and down the system to a steady state), when very short response and measurement times are involved. In addition, 2D clinostats are also subject to changes in forces other than gravity, including significant oscillating hydrostatic forces [30].

Due to these physical, technical, and operational limitations of simulated microgravity model systems, we conducted our investigations in real physical microgravity on parabolic flight missions (23rd DLR (Deutsches Zentrum für Luft- und Raumfahrt) parabolic flight campaign and 4th Swiss parabolic flight campaign) and a suborbital ballistic research rocket mission (TEXUS-51). However, the question of systematic comparability between gravity simulations and real gravity with respect to their effect on biological systems will remain open, as long as the fundamentals of coupling and transduction of gravity effects on the biological system are not sufficiently empirically known. This is because otherwise the physical consideration cannot be transferred to the appropriate biological verification context. Thus, this study attempts to contribute to a better understanding of the issue of coupling of genomic processes to gravity effects.

## 2. Results

We investigated the gene expression response to different gravitational environments in human Jurkat T lymphocytic cells in a multi-platform approach with a parabolic flight (23rd DLR Parabolic Flight Campaign), a suborbital ballistic rocket (TEXUS-51 mission), a 2D clinostat, and centrifuge experiments, in parallel with rigorous control experiments for excluding all possible other factors [30,31,33]. The analyses focused on the identification and distribution of gravity-responsive chromosomal regions (GRCRs) of differentially expressed genes (DEGs) that show intense differential expression in one direction.

Because the distribution of chromatin and chromosomal territories is non-random [54,55], we hypothesized that gravity-related DGE could be related to certain structural properties of the identified chromosome regions. Some chromosomal regulatory elements have been well known and will be analyzed in this study, including topology-associated domains (TADs) [56,57], lamina-associated domains (LADs) [58], and replication timing domains [59]. Furthermore, we analyzed the Giemsa stain cytobands and corresponding Alu sequences as an indicator of gene-rich and gene-poor chromosomal regions. These regions are correlated with chromatin folding, therefore influencing gene expression, and could represent a mechanosensitive element as well [60,61]. To directly analyze the involvement of structural alterations of the chromatin caused by altered gravity on DGE, we further performed a high-throughput chromatin conformation capture (Hi-C) experiment on the 4th Swiss Parabolic Flight campaign to be able to correlate conformational changes with the localization of GRCRs.

**Figure 1:**
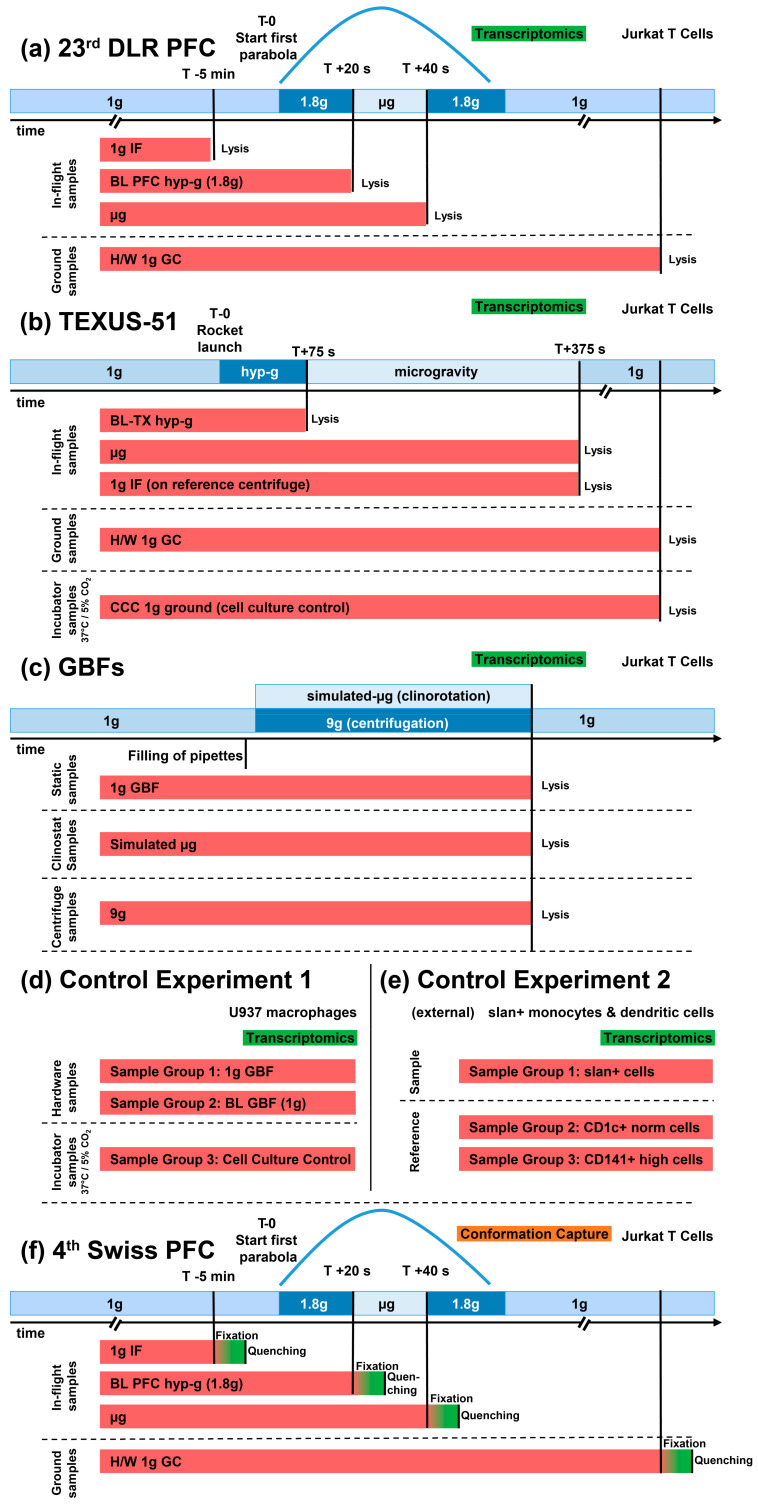
Experiment fixation scheme: overview over experimental conditions from which samples were acquired. Transcriptomics datasets are indicated in green, and high-throughput chromatin conformation capture (Hi-C) datasets are shown in orange. External datasets are labeled as such. (**a**) During the 23rd DLR (Deutsches Zentrum für Luft- und Raumfahrt) parabolic flight campaign (PFC) Jurkat cell samples were fixed shortly before initiating the first parabola (1gIF, compare Figure 1), at the end of the hypergravity phase (BL PFC hyp-g), and after the end of the subsequent first zero gravity phase. A ground sample which was inside the flight hardware but was not exposed to altered gravity was taken as a hardware ground control. (**b**) During the TEXUS-51 sounding rocket mission, the rocket payload was accelerated by two rocket engine stages for 75 s. At the end of the acceleration phase, the BL-TX hyp-g sample was fixed to monitor the hypergravity and vibrational effects. After another 300 s, the samples exposed to microgravity and samples spinning on an onboard 1× *g* centrifuge were fixed. A hardware 1× *g* ground control and a cell culture control were taken 15 min after the rocket launch. (**c**) During the Jurkat ground-based facilities campaign, the samples were exposed to vector-averaged gravity on a pipette clinostat spinning at 60 rotations per minute as a simulation of microgravity and to a 9× *g* pipette centrifuge to provide hypergravity. The samples were fixed after 5 min. A static sample as a control was fixed in parallel that was aspirated to pipettes but was only exposed to vibration at the clinostat base plate. (**d**) Control groups were acquired during the U937 ground-based facilities campaign. The BL sample was fixed immediately after being filled into pipettes, and the 1× *g* ground-based facility (GBF) samples stayed in pipettes at 1× *g* for 300 s before fixation. A cell culture control was taken in parallel. (**e**) External control experiment 2 (GSE98694) based on human immune-derived cells that were analyzed on the same microarray (AffyMetrix HTA2.0) as all other experiments. Three sample groups were included in the datasets, slan+ cells that were analyzed in the original study, CD1c+ reference cells (sample group 2), and CD141+ reference cells (sample group 3). (**f**) During the 4th Swiss Parabolic Flight Campaign (Swiss PFC), the samples were acquired from the same conditions as from the 23rd DLR PFC campaign. Here, chromosome conformation capture samples (Hi-C) were generated by crosslinking the samples with 3% formaldehyde and subsequently quenching the crosslinking reaction after a 3 min incubation time with glycine.

In addition, internal (see section data availability) and external reference datasets [62] were included for comparisons. These datasets were obtained from other immune cell samples that showed DGEs due to effects that were measured using the identical microarray model but did not result from altered gravity. Detailed sample conditions are described in Figure 1 and Table 1.

Prior studies on the DGE of human immune cells revealed a complex transcriptional response to short-time hypergravity and microgravity that involved hundreds to thousands of significantly DEGs [31,34]. Between 20 s and 75 s of hypergravity and between 20 s and 5 min of microgravity, a rapid adaptation was identified: previously upregulated genes appeared non-responsive or mostly downregulated, previously downregulated genes appeared non-responsive or mostly upregulated, and further new DEGs emerged [34]. Therefore, the temporal conservation of transcriptional response did not appear on the level of single genes.

**Figure 2:**
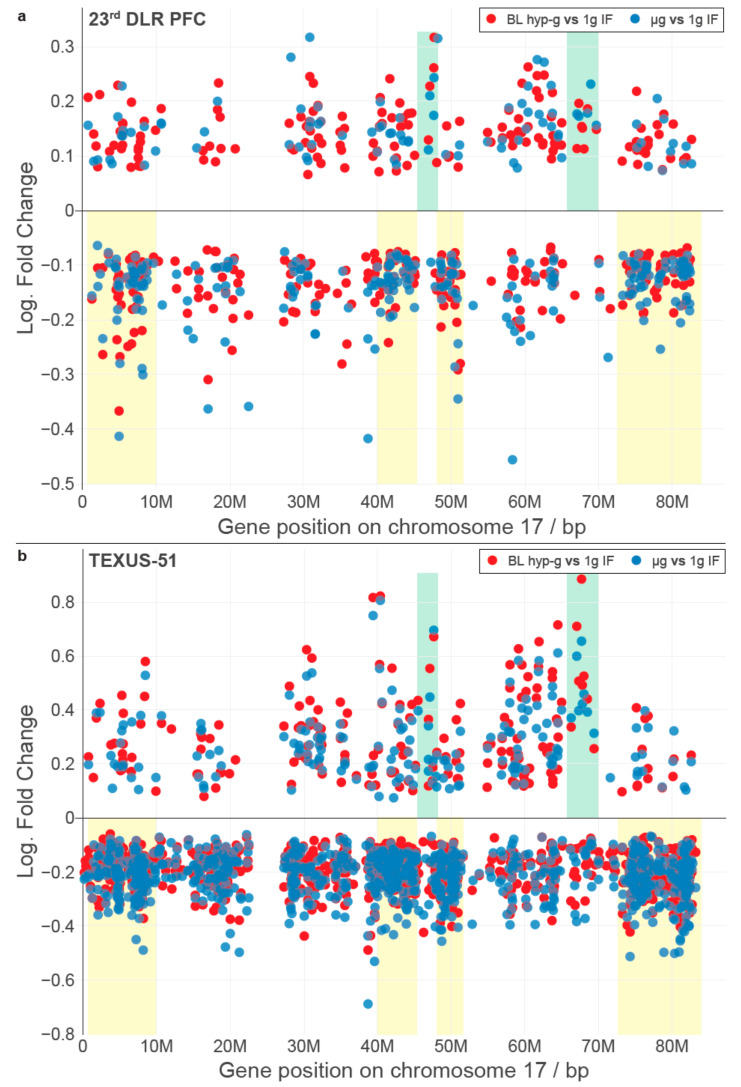
One-dimensional (1D) projection of significantly differently expressed genes (false discovery rate-adjusted *p* value < 0.05) along chromosome 17 of the selected altered gravity datasets. Regions that show an accumulation of downregulated genes in all datasets are indicated in yellow, and regions that show strongly upregulated genes with only a few to no downregulated genes in the same cluster are indicated in green. (**a**) Comparisons for hypergravity and microgravity from the 23rd DLR PFC; (**b**) comparisons for hypergravity and microgravity from the TEXUS-51 sounding rocket campaign.

We wondered if there were other conserved elements in the transcriptional response towards the altered gravity of different lengths besides single genes. In a recent publication, we could identify that short-time gravity-related DGE was distributed almost equally over all chromosomes but showed a strong bias in terms of up- versus downregulation on certain chromosomes [63]. Based on this, we asked if the distribution of DEGs among chromosomes could be conserved between different altered gravity comparisons. We exemplarily plotted all significantly differentially expressed transcripts (false discovery rate-adjusted *p* values < 0.05) for both the hypergravity and microgravity comparisons of the TEXUS-51 study and the 23rd DLR Parabolic flight campaign on chromosome 17 (Figure 2; for all other chromosomes see Supplementary Figure S1). We noticed that there were certain areas in the one-dimensional (1D) projection of the chromosome with a high density of DEGs that mostly carried downregulated genes, not only in the two comparisons of one dataset, but also for the other dataset (Figure 2, yellow boxes). Further, we noticed certain regions that seemingly carried significantly upregulated genes with unusually strong fold changes with no to only little downregulated genes in the same area (Figure 2, green boxes). These regional clusters of DEGs were called GRCRs.

Intrigued by the first observations, we analyzed if GRCRs consistently appeared in other datasets and if they co-localized specifically for gravity-related datasets, i.e., if GRCRs were a conserved element in altered gravity transcriptomics instead of single genes. To test this hypothesis, we developed a GRCR analysis methodology that sectioned the 1D projection of gene expression on all chromosomes, tested whether such a section was a GRCR and quantified how strongly potential GRCRs were correlated between different experiment comparisons. The resulting measures were two correlation coefficients per pair of comparisons, with one average expected correlation coefficient and one actual correlation coefficient. The larger the difference between these two, the more the DGE was driven by GRCRs that were conserved between both comparisons. A small difference or no difference implied that potential overlapping regions between two comparisons were likely generated by pure chance (random drawing). The latter could for example be the case if the same genes were differentially expressed between two comparisons, and therefore, the conservation between such comparisons would not be on the level of regions, but of single genes. A detailed derivation, confirmation, and testing can be found as Supplementary Text S1, details in Supplementary Figures S5–S9 and S11, and Supplementary Table S2.

### 2.1. Inter-Experiment Correlations Could Be Explained by Conserved GRCRs

**Figure 3:**
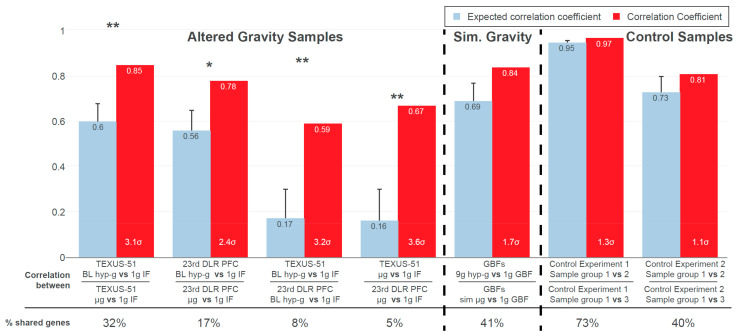
Results from gravity-responsive chromosomal region (GRCR) analysis: average expected correlation coefficient and first standard deviation (blue, error bars) vs. actual Spearman correlation coefficient (red) of selected comparisons. Results are grouped into those where the transcriptional effects emerged from different gravity conditions and those that did not. The relative difference is indicated in standard deviations at the bottom of the columns. Significantly different samples with inter-experiment correlation coefficients of >2σ are indicated with *, and highly significantly different samples with inter-experiment correlation coefficients of >3σ are indicated with **. The larger the relative and absolute difference in correlation strength between the expected and the actual dataset, the more a comparison deviates from the average distribution differential gene expression. Below, the percentage of genes that are shared between the two compared differential expression sets out of all genes that contribute to the averaging bins are listed. The strongest absolute and relative difference in correlation can be observed for inter-experiment comparisons for altered gravity contrasts, which also share the smallest number of genes.

Figure 3 shows the results from the GRCR analysis for the correlations between gravity-related comparisons within one experiment (intra-experiment correlation) and between different experiments (inter-experiment correlation), for a GBF experiment with simulated altered gravity and for two reference datasets. The latter served as negative controls, with the same type of transcriptomics microarray that showed differential expression but was not associated with altered gravity. 

At first, the comparisons within an experiment were correlated, i.e., the hypergravity versus control comparison against the microgravity versus control comparison. The actual correlation coefficients of 0.85 (TEXUS-51) and 0.78 (23rd DLR PFC) (Figure 3, red bars) indicated that sections highly correlate between the comparisons. Intra-experiment correlation coefficients for real altered gravity conditions declined by 0.25 (TEXUS-51) and 0.22 (23rd DLR PFC), i.e., 3.1 σ and 2.4 σ in relative numbers, respectively, between the actual and expected datasets; 3 σ corresponds to a likelihood of generating a stronger correlation under the null hypothesis (by random permutation) of 0.27%. Therefore, the observed values were highly significant compared to the null hypothesis. The high intra-experiment correlations of altered gravity conditions can thus be partly explained by specific GRCRs and partly by conservation on the level of single genes. 

Next, DGE was compared between the two altered gravity experiments. Inter-experiment correlations for the two hypergravity vs. normal gravity datasets and the two microgravity vs. normal gravity datasets demonstrated a strong difference in correlation of 0.42 (hypergravity) and 0.51 (microgravity), i.e., 3.2 σ and 3.6 σ in relative numbers, respectively. Therefore, these two inter-experiment correlations can profoundly be explained by conserved GRCRs, but not by the general distribution of fold changes respectively a conservation on the level of single DEGs. 

Contrarily, the correlation decreased only marginally in the two control experiments. Thus, their structure of DGE can be fully explained by the general distribution of fold changes respectively conservation on the level of single DEGs. They did not show the characteristics of GRCRs and therefore behaved fundamentally different compared to real altered gravity datasets. Interestingly, the correlations between the simulated (vector-averaged) microgravity and centrifuge-induced hypergravity condition in our study had only a weak decrease, showed only a minor GRCR dependence and behaved more like the control datasets.

Additionally, the number of shared genes that contributed to the correlation calculations was evaluated. They indicated whether the correlation was driven by identical genes or by entire regions with different significant genes per dataset. The hypergravity and microgravity inter-experiment comparisons had the lowest percentage of shared genes used for bin average calculations, despite their significant actual correlation. This further underlined that for these dataset comparisons, the correlation was not driven by pairs of genes but by the same GRCRs containing different significantly expressed genes in each comparison. Control experiment 1 had the highest percentage of shared genes (73%), but the lowest difference in correlation. A detailed structural analysis on the level of DEGs can be found in Supplementary Text 1.

### 2.2. Linear Modeling of DGE for Different Chromatin Motifs Reveals TADs and Chromosome Length/Ends as Underlying Structures

The next step was to better understand what drives the GRCR effect. As previously described, the effect was not driven by shared genes but apparently by genomic localization. We could further exclude functional gene sets as the underlying explanation (Supplementary Text S2, Supplementary Tables S3 and S4) and performed an in-detail analysis on the potentially functionally relevant gene sets GO_TRANSCRIPTION_REGULATOR_ACTIVITY, GO_BIOLOGICAL_ADHESION, and GO_G_PROTEIN_COUPLED_RECEPTOR_SIGNALING_PATHWAY, which did not yield a significant gravity-specific clustering of groups of genes that emerged together (Supplementary Figure S12). Additionally, a direct coupling of gravitational forces to the nucleus has been previously postulated for short-term altered gravity effects [30,34]. Therefore, an involvement of structural chromatin elements was assessed. We tested different chromatin structure elements that have been previously reported to influence gene expression to determine possible correlations with the observed GRCR effect. The rationale behind this was to identify structures that could play a mechanistical role in the observed DGE, which could shed light on underlying mechanisms. Chromatin bears several structural elements of different sizes: 1. chromosome length; 2. chromosome ends vs. middle part; 3. density of Alu domains; 4. early vs. late replication domains; 5. Giemsa stain chromosome cytobands; 6. TADs; 7. LADs; 8. a combinatory model between chromosome length and chromosome ends; and 9. a combinatory model between chromosome lengths, chromosome ends, Alu domains, and replication domains. Many of them are correlated (such as Alu domains, replication timing, and cytobands) but are measured by different effects and are not fully identical; therefore, all these elements were tested.

**Figure 4:**
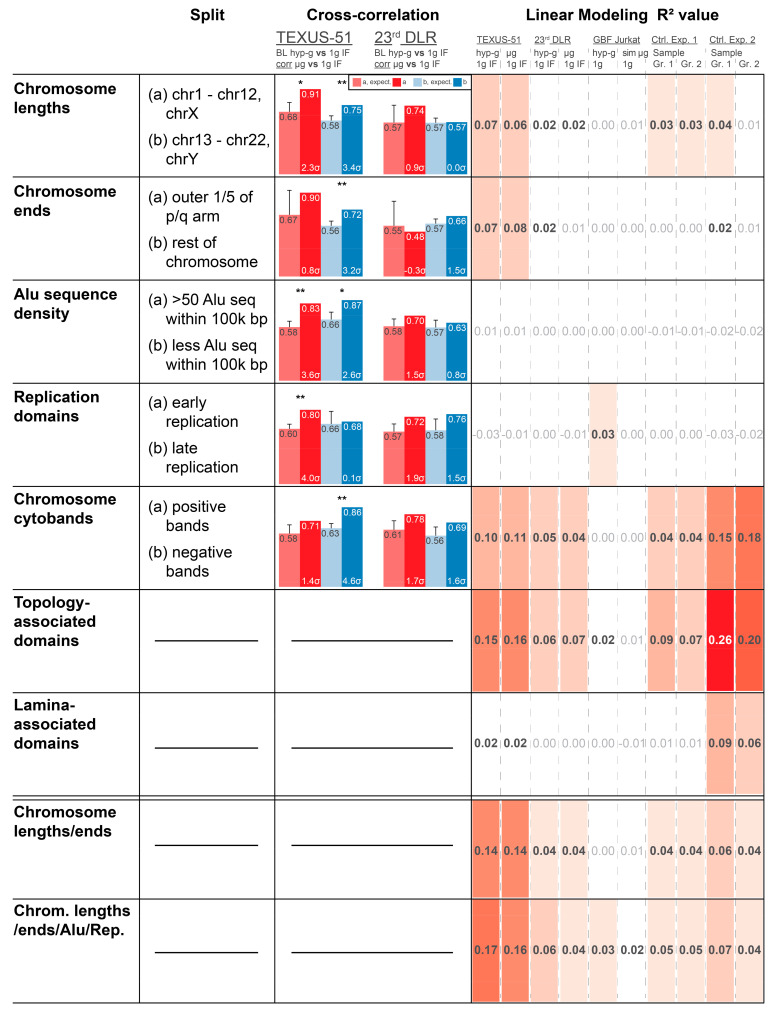
Model hypothesis testing results on the analysis of different potential underlying DNA structures that could be involved in the observed differential gene expression patterns. Each row represents one structural element, and the last two rows are combinatory models. The first column “split” describes the structural element-based rule by which the top 2000 differentially expressed genes are separated into two groups. For both subsets, a correlation of regional trends analysis was performed independently. The following columns show the results of the correlation of regional trends analysis, the Spearman correlation coefficient for the gravity-related intra-experiment comparisons, TEXUS-51 hyper gravity vs. 1× *g* inflight (1gIF) compared with microgravity vs. 1gIF and the same comparison for the 23rd DLR PFC experiment. Significantly different samples with inter-experiment correlation coefficients of >2σ are indicated with *, and highly significantly different samples with inter-experiment correlation coefficients of >3σ are indicated with **. A difference in behavior between split sets (a) and (b) indicates an underlying structure in the data represented in those categories. For topology-associated domains, lamina-associated domains, and the mixed models, no categorial split into two categories could be defined that provides enough bins per category to be able to perform this analysis. The column “linear modeling” provides the average R2 predictive strength for the logFC of genes of linear models based on the underlying DNA structures. Fits were performed on all used data groups separately (columns) and performed independently on datasets including the top 2000 genes. Results for the top 5000 genes and all 16,800 uniquely mapping genes can be found in Supplementary Materials. Due to the very rudimentary linear models, no high R2 values are expected. The scale ranges from 1 (perfect predictive strength) over 0 (no predictive strength) and can be arbitrarily lower (prediction worse than the random drawing of predictions). Color coding is from dark red (highest predictive strength for any hypothesis for all experiments per dataset of top n regulated genes) to white (0.01 or below). Topology-associated domains had a high predictive power for control experiment 2 and therefore define the upper limit of the relative color scale.

To assess the influence of chromatin structures and structure combinations, two analyses were performed. Firstly, actual vs. expected average correlation coefficient analyses were performed as previously described. However, the datasets were split into two separate subsets that were analyzed independently, based on the analyzed structure (e.g., separate approaches for the subsets containing Alu-domain-rich and Alu-domain-poor regions). A clear correlation difference for one subset, but not for the other, would give an indication that correlation coupling depends on the structure analyzed. Secondly, the predictive strength of a model based on a certain feature was analyzed. The R^2^ value of a model represented how well a feature determined the fold changes of genes of each dataset from 0 (no predictive strength) to 1 (maximum predictive strength). This approach benchmarked how much DGE was dependent on the structural elements analyzed (Figure 4 for top 2000 DEGs; Supplementary Figure S2 for top 5000 AND 16800). 

The influence of chromosome length was evaluated with two sets, long chromosomes 1–12 and X vs. short chromosomes 13–22 and Y (because the largest difference in length in the middle range is between chromosome 12 and 13). For TEXUS-51, the correlation coefficients were not different between these two sets, but for 23rd DLR PFC, the longer chromosomes had lower correlation coefficients, both before and after shuffling. The linear model revealed a structure-mediated significance in the TEXUS-51 data for all three modeling levels (2000, 5000, and 16,800 genes), which was nearly not present in the 23rd DLR PFC data, the two control experiments, and the ground-based facility (GBF) experiments (Figure 4). The model for chromosome ends was based on a split between the outer 1/5 of the p/q arms and the rest of each chromosome, resulting in a clear correlation coefficient difference for the TEXUS-51 and 23rd DLR PFC experiments. Again, another simplistic linear model, based only on 3 independent values (outer p, outer q, and rest) had a significant predictive strength for TEXUS-51 at 2000 and 5000 genes. Therefore, chromosome lengths and ends represent a potentially structural explanation of the observed differential gene expression in the TEXUS-51 experiments.

Both the density of Alu sequences and early vs. late replication domains did not exhibit strong effects on cross-correlation and predictive strength for linear modeling, although Alu-rich sequences demonstrated a slightly stronger correlation than Alu-poor regions. The linear models based on Alu sequence density and replication domains are considerably more simplistic than the aforementioned models and only consist of one numeric variable, which leads to models that are potentially too reductionistic to exhibit predictive strength. None of these structures on their own were dominant candidates to explain the observed behavior.

The cytoband analysis revealed less correlation for positive chromosome cytobands compared to negative cytobands for the 23rd DLR PFC. In addition, there was a robust predictive model strength for all three-gene dataset levels both for the TEXUS-51 and for the control experiment 2 data. A weak predictive strength was found in the 23rd DLR PFC data. Therefore, light and dark cytobands may be partially relevant to the observed DGE signal. Although this feature also exhibited considerable predictive strength in control experiment 2, control experiment 2 did not show a GRCR effect that differed from average expectation. This shared cytoband feature might also contain genes that contribute to the part of the correlation strength that does not differ between the actual and expected GRCR effects in mixed models.

A more complex linear model was developed for analyzing the involvement of TADs. A considerable predictive strength was found for control experiment 2 and the TEXUS-51 datasets, whereas a medium strength was also detected in every other dataset, except for the GBF data. The high values for the control experiments are intuitive, since TADs have already been proven as a central general player of gene regulation, which acts independently of altered gravity. Therefore, the R^2^ values serve as reference points of the maximum predictive strength under our linear modeling approach. In contrast, LADs only showed a good predictive strength for control experiment 2 and therefore are less likely to influence altered gravity effects on DGE.

A linear combinatory model on chromosome ends and lengths had an enhanced predictive strength for TEXUS-51 and was almost as strong as the TAD-based model. Therefore, these two structural components combined are strong candidates that explain the observed effects for TEXUS-51 and potentially also for 23rd DLR PFC data. Adding Alu and replication domains as factors improved the predictive strength, resulting in high R^2^ numbers that even slightly outcompete those from the superiorly complex TADs model. Particularly, of interest is the fact that the predictive strength for the control experiments is much weaker for these mixed models, clearly indicating the involvement of chromosome structure (ends of chromosome arms and different chromosomes) and a potential involvement of the Alu domain density and replication domains in DEGs under altered gravity.

### 2.3. DGEs and Locations of Chromosomes 18 and 19

**Figure 5:**
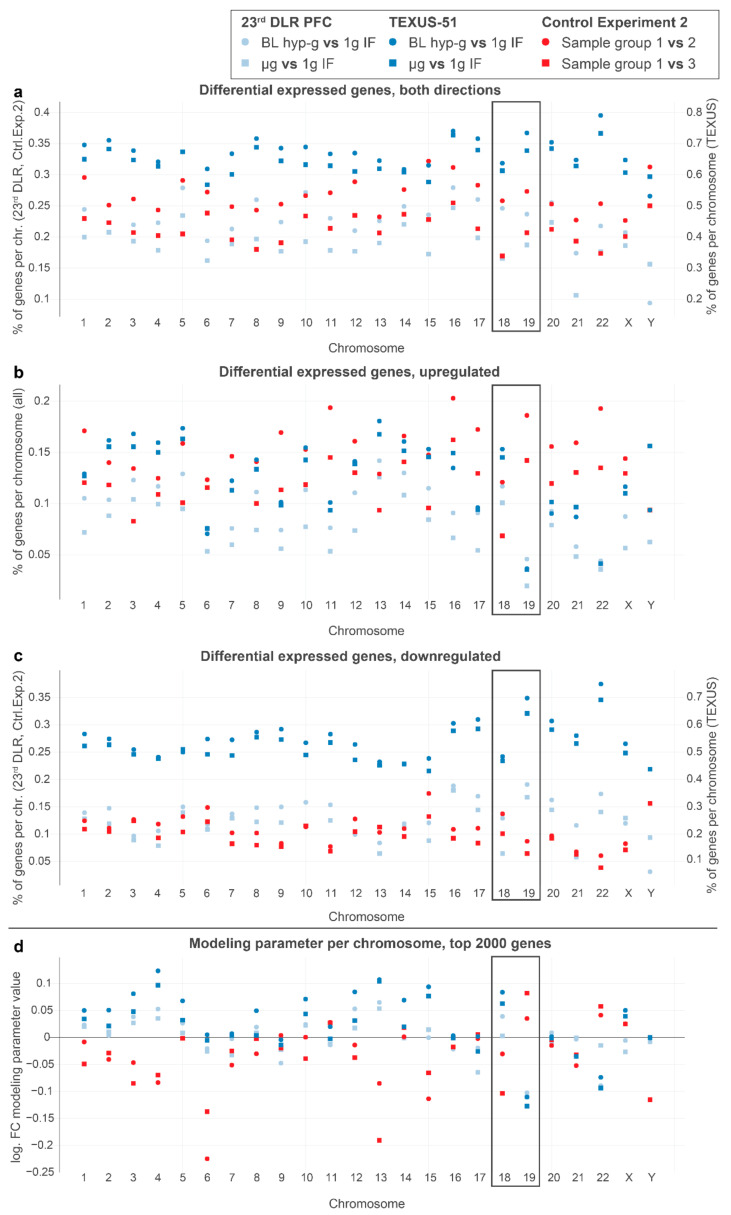
Pinpointing differential gene expression to single chromosomes. (**a**–**c**) The percentage of differentially expressed genes, defined as genes with uncorrected *p* of <0.05 divided by the number of all measured genes per chromosome for all differentially expressed genes (**a**), upregulated genes only (**b**), and downregulated genes only (**c**). (**d**) Elastic net modeling parameter (analysis from Figure 3) describing the overall log fold change for the predicted values that is assumed for the entire chromosome in the chromosome length linear model based on the top 2000 genes. Chromosomes 18 and 19 are highlighted as reference points with known geometry for Jurkat cells. Real altered gravity conditions are marked in blue, and control experiment 2 conditions are indicated in red.

In human Jurkat T cells, chromosome 18 and chromosome 19 are the most studied in terms of nuclear localization and can therefore be utilized for model testing. Chromosome 19 (chr19) is likely located close to the nuclear center, whereby chr18 is located close to the nuclear membrane, representing a transcriptionally more repressive environment than the inner areas of the nucleus [64]. Upon altered gravitational force, we expected that gene expression changes were associated with chromosome localization, as demonstrated previously. Due to their distinct locations, chromosome 19 could only move outwards into repressive areas, and chromosome 18 could only move inwards into expressive areas of the nucleus. Thus, we expected gene expressions in the outer chromosome 18 and in the inner chromosome 19 responding in the opposite directions, with more specific upregulation for chromosome 18 and more specific downregulation for chromosome 19 under altered gravity. We therefore analyzed the percentage of DEGs per chromosome for different DGEs (Figure 5a–c) and the overall estimated log fold change for the entire chromosome in the aforementioned chromosome length linear model (Figure 5d).

Indeed, we detected gene expression upregulation in chromosome 18 and downregulation in chromosome 19 for all alterations of real gravity conditions. The log-fold change modeling parameters for chromosome 18 exhibited a positive fold change and that for chromosome 19 presented a negative fold change, which was unique for real altered gravity conditions. These findings were therefore consistent with published studies on chromosome location. The effects could not be detected in “control experiment 2” and other controls (Supplementary Figure S3) or in simulated altered gravity samples.

### 2.4. Chromatin Conformation Capture Analysis Reveals a Direct Involvement of Chromatin Architecture in Gravity-Responsive Region Transcriptional Effects

**Figure 6:**
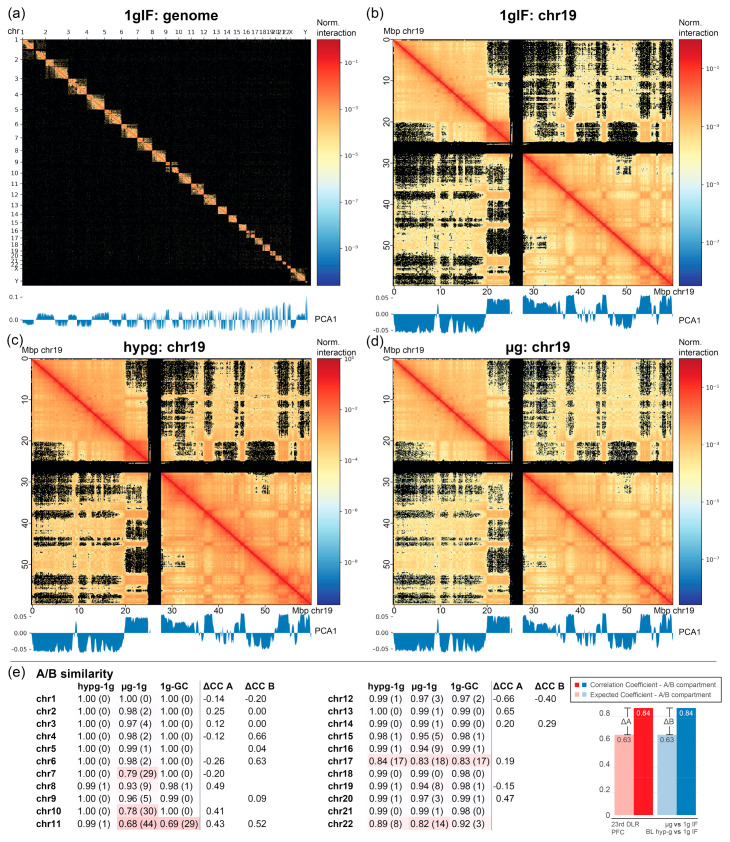
Chromosome conformation capture in altered gravity. (**a**) Interaction map of the entire human genome of Jurkat human immune cells during the 4th Swiss PFC. Chromosomes and chromosome compartments appear as rectangles. Pearson interaction scores are given. The A/B compartment eigenvector score as a measure of eu-/heterochromatin compartmentalization is given below. (**b**–**d**) Interaction map of chromosome 19 and A/B compartmentalization for the three flight phases (i.e., 1× *g* inflight reference, hypergravity, and microgravity). No larger structural change of A/B compartments could be identified. (**e**) Quantification of the A/B similarity among different flight conditions. Given is the medium and the first standard deviation of the Pearson correlation coefficient of the A/B calling tracks between the samples of two flight conditions. Hypg-1g represents the in-flight changes between normal gravity and hypergravity, µg-1g represents the effects of microgravity, and 1g-GC indicates the flight vs. ground reference control. Additionally, the differences in actual and expected correlation coefficients for the two 23rd DLR PFC transcriptomics samples are given for the A and B compartment subsets per chromosome if applicable (compare Figure 4 subset testing). The score is explained in the diagram on the right.

The prior analyses indirectly provided evidence on the involvement of chromatin structure in the early transcriptional reactions in response to altered gravity. We therefore wanted to gain insights into the effects of altered gravity on chromatin conformation. The question arose if gravity could alter the conformation of the chromatin and therefore led to DGE. To directly test our hypothesis, a Hi-C experiment was performed with Jurkat T cells during the 4th Swiss Parabolic Flight campaign in different gravity environments. Samples were acquired during the 1× *g* inflight phase (1gIF) and the hypergravity (hypg) and microgravity (µg) phase of the first parabola, as well as on the ground as controls (GC), in three biological replicates for each condition. Intrachromosomal and interchromosomal interactions were mapped for all samples (Figure 6a). For all chromosomes, A/B compartments were determined representing chromatin states especially associated with euchromatin vs. heterochromatin. No fundamental compartmentalization changes on the whole chromosome scale could be detected between the different conditions (Figure 6b–d; representative visualization of chromosome 19). 

Next, the replicates were leveraged by calculating the Pearson correlation coefficient average and standard deviation among all samples from different conditions for all comparisons. This was compared to the difference of actual vs. expected correlation coefficients on the datasets split into two separate subsets, based on the A/B compartments per chromosome (similar to the cross-correlation analysis in Figure 4). This was not possible for every chromosome, since bins did not contain the required number of 10 genes in some chromosomes. If a chromosome showed significant differences in A/B compartment similarity for the hypergravity and/or the microgravity comparison, but not for the 1gIF vs. GC, this would indicate a gravity-dependent effect. Additionally, if this chromosome had a correlation coefficient for one compartment that was different from the other compartment, this would likely indicate a structural involvement of A/B compartmentalization in DGEs. Chromosomes 7, 10, and 22 displayed an A/B dissimilarity for altered gravity comparisons, but not for 1g comparisons. Chromosomes 2, 4, 6, and partly 12 demonstrated the fundamental differences between expected vs. actual correlation of gravity-responsive chromosomal site transcription between A and B compartments. However, no chromosome fulfilled both. Differences in transcription bin averages could be due to the A/B compartmentalization but were not associated with structural changes in A/B compartments. 

**Figure 7:**
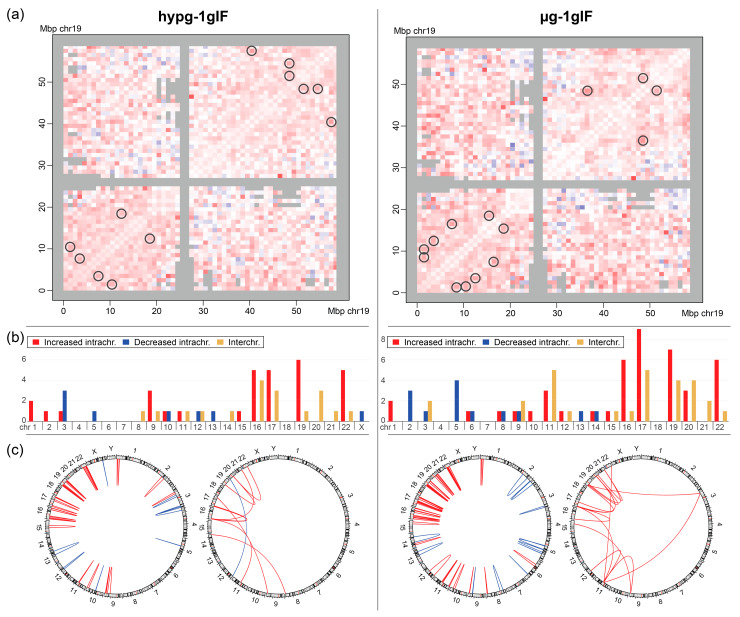
Differential chromosome conformation captured to analyze the effects of altered gravity. (**a**) For the comparisons between hypergravity and 1gIF and between microgravity and 1gIF samples, the Hi-C interaction maps were analyzed for significantly different interactions. Here, differential interaction is displayed for chromosome 19. Colors represent log fold changes, independently of signal strength. Bin pairs that show an increased interaction are highlighted in red, and decreased interaction is highlighted in blue. Bin pairs that show the most statistically significant interaction between conditions are circled. (**b**) Distribution of significantly different bin pairs over all chromosomes. Intrachromosomal bin pairs are stratified by increased and decreased interaction. For interchromosomal gene pairs, both contacts are listed separately; therefore, for one interaction, both chromosomes appear in the plot. Interchromosomal pairs are not split by increased and decreased interactions, since all of them are increased except for one interaction (compare **c**). (**c**) Significantly different interactions for intra- (left circus plot) and interchromosomal (right circus plot) interactions. Increased and decreased interaction is color-coded. Intrachromosomal interactions that have bin pairs close to each other on the chromosome strand appear as strong single lines instead of arches.

Furthermore, interacting bin pairs from the Hi-C datasets were systematically assessed for significantly differential interactions (Figure 7a; representative visualization of differences for chromosome 19). The bin pairs of 1Mb were separated by (strong signals) intrachromosomal interactions and (weak signals) interchromosomal interactions and then tested separately. For the hypergravity vs. 1gIF comparison, 39 intrachromosomal interactions (at the lowest FDR (Benjamini Hochberg false discovery rate) cutoff level of 0.25) and 9 interchromosomal interactions (at the lowest FDR cutoff level of 0.33) were identified as slightly significantly differential interactions. For the microgravity vs. 1gIF comparison, the differences were more robust with 56 intrachromosomal interactions (at the lowest FDR cutoff level of 0.1) and 15 interchromosomal interactions (at the lowest FDR cutoff level of 0.2). The full list of significant bin pairs can be found in Table S1.

The stratification of bin pairs by chromosome yielded a highly non-random distribution (Figure 7b). Generally, small chromosomes (chr16–22, except for chr18) showed increased intra- and interchromosomal interactions in altered gravity, whereas larger chromosomes had more balanced responses with more chromosomes, showing decreased intrachromosomal interaction, such as chromosomes 2 (only µg), 3, and 5. This overall trend was more pronounced for µg vs. 1gIF comparison. Interchromosomal interactions (all increased except for chr12 vs. chr20 for hypg vs. 1gIF) were also almost exclusively significantly different at the smaller chromosomes. When plotting the locations of interacting bins against each other, this trend became more visible (Figure 7c). For the intrachromosomal interactions, the small chromosomes 16, 17, 19, 20, and 22 had high numbers of differentially interacting bins compared to the larger chromosomes, with mostly increased interactions. In contrast, larger chromosomes showed more decreased interactions, with a localization preference towards the chromosome’s q arms for µg vs. 1gIF. For the interchromosomal interaction, chromosomes 19 and 11 interacted with a region in the p arm of chromosome 3 in microgravity (Figure 7c; most right circos plot), in hypergravity, there was one increased interaction between chromosomes 8 and 14 (Figure 7c; second from the left circos plot). All other interchromosomal interactions appeared between the chromosomes chr9 and chr22 with a focus on chr16 and chr17 in hypergravity, and chr19, chr17, and chr11 in microgravity.

**Figure 8:**
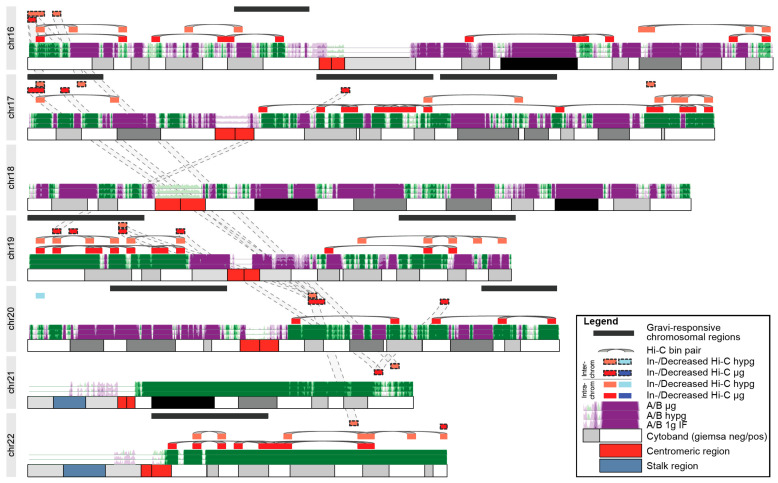
Overlap between gravity-responsive transcriptional regions from clustering correlation analysis (both 23rd DLR PFC comparisons had a transcriptional binning average for these sites) and significant differential Hi-C bin pairs. Hi-C bin pairs are linked by grey curves. The A/B compartments are highlighted for all three in flight conditions, with 1gIF, hypg, and µg in ascending order. Here, the chromosomes with the majority of differential interaction sites are shown (chr16–22). The full genome is shown in Supplementary Figure S10. The non-randomness of Hi-C bins towards differential transcription clusters over the entire genome was assessed with a Fisher’s exact test as 4.7 × 10^−5^ for hypg vs. 1gIF and as 1.3 × 10^−9^ for microgravity vs. 1gIF.

Finally, we analyzed further to explore whether these systematical effects of chromatin conformation changes could be the underlying explanation of GRCRs identified by DEG datasets. We focused on small chromosomes (chr16–22), since they bear the majority of differential Hi-C interactions. When plotting the A/B compartmentalization for these chromosomes, no particular preference of the significantly differential Hi-C bins for one type of compartment could be identified. We then plotted the bins containing the identified GRCRs for the 23rd DLR PFC datasets against bin pairs with significant differential Hi-C intra- and interchromosomal interactions (Figure 8 for chr16–chr22; see Supplementary Figure S4 for the entire genome; pairs of significantly differential bin pairs were highlighted by grey lines; all significant bin pairs in Supplementary Table S1). Therefore, intrachromosomal pairs resembled two loci that showed increased (or decreased if blue) interaction in hypergravity or microgravity. On chr16–chr22, we identified nine GRCRs. Only one of them on chr20, did not overlap with any differential Hi-C bins. Out of the 39/56 intrachromosomal differential bins for hypergravity/microgravity, approximately half of them (19/32) localized within gravity-responsive transcriptional regions, while these GRCRs spanned significantly less than half of the chromosomes.

This results in a highly significant bias of localization of differential Hi-C interaction towards GRCRs for both comparisons (Fisher’s exact test *p*-value of 4.7 × 10^−5^ for hypg vs. 1gIF and 1.3 × 10^−9^ for µg vs. 1gIF). Additionally, some of the intrachromosomal bins that did not localize within gravity-responsive transcriptional regions were close to or overlap with interchromosomally interacting bins that were connected to gravity-responsive transcriptional regions on a different chromosome. For example, the outer p arm of chr16 interacted with the gravity-responsive region on the outer p arm of chr17 (Figure 8). The outer p arm of chr17 was a region with significantly increased interaction in hypergravity that colocalizes with a GRCR. There were additionally 2 bins in hypergravity and 3 bins (2 + 1) in microgravity that were within the GRCR and demonstrated increased interchromosomal interaction. At the start of the GRCR, there was one particular bin that was significant on all three of these layers that interacts with the beginning of chr16. Another cluster of interchromosomal and intrachromosomal bins on chr16 showed an increased interaction in both hypergravity and microgravity. They were not covered by a GRCR, but due to the interchromosomal bin linkage to chr17, were likely in proximity to the GRCR on chr17.

In conclusion, the highly significant association of changes in intrachromosomal interactions and the interconnectivity by interchromosomal interactions indicated an association of structural effects with gene expression of GRCRs.

## 3. Discussion

In recent decades, it has become evident that mechanical forces are captured and transduced by cellular [10,40,41] and nuclear architecture [43,44], leading to changes in nuclear plasticity, chromatin organization and accessibility, and subsequently gene expression [36,65]. While cellular structures in the Earth’s gravitational field are under permanent gravitational force, it has been shown that an alteration in the gravity environment, i.e., microgravity or hypergravity, has profound effects on the cellular and molecular levels, causing cell shape changes as well as cytoskeletal alterations [10,26,39,66,67,68].

Currently, two hypotheses could explain how mechanical stresses such as shear forces or changes in the gravitational vectors are transmitted from the exterior inside the cell and the nucleus to induce gene expression changes: (1) transformation of mechanical force through biochemical signaling by chemical signal propagation, such as calcium signaling (several, up to 10 s); (2) direct mechanical force propagation via a highly connective intercellular structure like the cytoskeleton (milliseconds to a few microseconds) [69]. Mechanical forces are perceived at and across the cell membrane and induce signaling pathways inside the cell involving the cytoskeleton [38,69]. Force transmission through the cytoskeleton to the nuclear envelope has been shown [70], resulting in nuclear morphology remodeling and affecting orientation, three-dimensional (3D) radial position, and intermingling of chromosome territories (CTs) [48], and chromatin condensation [71], accompanied by DGE patterns [72]. One important component of this force transduction across the nuclear envelope is the linker of nucleoskeleton and cytoskeleton (LINC) complex [46]. It consists of inner and outer nuclear membrane proteins and has a physical link to the cytoskeleton [73]. The LINC complex binds to nuclear lamin proteins located on the inside of the nuclear membrane. Lamins can interact directly with chromatin or indirectly via histones or associated proteins [74]. When mechanical forces transmitted via the cytoskeleton reach the nucleus, lamin proteins can react with conformational changes [75]. The interaction of lamin proteins with transcription factors can lead to gene expression changes impacting proliferation, differentiation, and apoptosis [74,76]. Additionally, at specific locations, the chromatin comes in close vicinity to the nuclear lamina itself where repressive heterochromatin with low gene expression levels predominantly occurs [77]. These specific regions are called LADs. It has been shown in mammals that the lamin-bound heterochromatin organized in LADs represents up to 40% of the genome [75,78].

**Figure 9:**
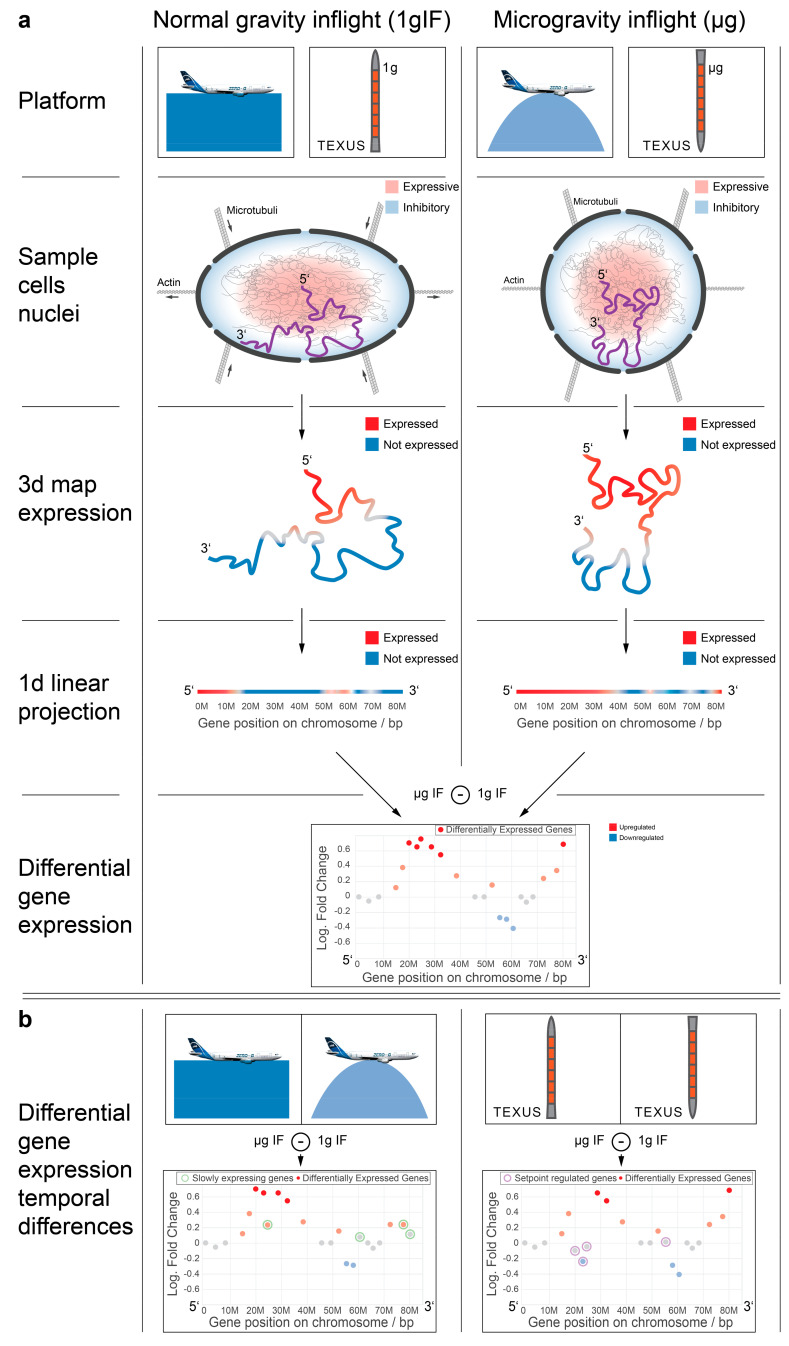
Summary of the proposed hypothesis. (**a**) The effects of altered gravity on cellular gene expression are proposed to have a mechanical nature. Cells on different platforms are exposed to both normal gravity and microgravity. During the normal gravity, the forces acting by gravity slightly compress the nucleus in the vertical axis, therefore shortening it in the vertical axis and prolonging it in the horizontal axis. In microgravity, these forces are no longer present, and the nucleus takes a more roundish shape. Resulting from different nuclear shapes, the chromatin has to take different orientations, depending on the shape. An exemplary chromosome, highlighted in purple, takes different conformations, depending on the platform flight phase. Different areas of the nucleus are known to exhibit different expressivities, the outer areas are rather repressive, and the inner areas are rather expressive. This leads to different expression strengths for different genes, depending on where the gene is located on the chromosome and therefore in the nucleus. When projecting this three-dimensional (3D) to one-dimensional conformation, the distinct expression/repression pattern also becomes visible in one dimension. Because of the conformation change upon altered gravity, there are regional differences in expressivity, if regions are no longer in same areas. When subtracting both patterns from each other during transcriptomics studies, this leads to regional patterns of fold changes distributions of differentially expressed genes. (**b**) Differential gene expression datasets from different platforms are not equal on the gene level, since slowly expressing genes only move to their distinct regional level after a longer period of time (not given during a parabolic flight; green circles), and next to mechanogenetic effects, further gene regulatory effects will influence the expression patterns, for example setpoint regulation bringing back upregulated genes to target levels after a while (purple circles). This explains why differential expression sets from similar conditions on different platforms differ a lot, even if they have the same underlying effect. This disturbance effect on the level of single genes can be compensated for by the GRCR analysis described here.

In our gene expression analysis, we reported the existence of chromosomal regions that demonstrated accumulation of DGE and were sensitive to changes in the gravitational force (GRCRs). In order to prove that these co-regulated chromosomal regions identified in one-dimension depend on 3D chromosomal conformational changes and that these conformational changes instantaneously occur upon gravitational changes, we performed genome-wide chromosome conformation capture (Hi-C) analyses. Hi-C is a recently developed method to study the 3D architecture of the whole genome [79,80] and to identify stimulus-induced conformational changes [81,82]. We investigated whether the exposure of cells to short-term hypergravity and microgravity leads to significant changes in the chromosomal architecture. We firstly investigated the contents of A and B compartments representing eu- and heterochromatin distinguished by Hi-C. Our analyses revealed no major changes for the comparisons of 1gIF versus ground control, hypergravity versus 1gIF, and microgravity versus 1gIF (Figure 6b–e and Figure 9). The overall stability of A and B compartments is not surprising, because the ultrashort stimuli of 20 s altered gravity during a parabolic flight are most likely not long enough to cause major changes in eu- and heterochromatin distribution. Ray et al. showed that, even after 30 min of the heat shock stimulus, only subtle changes in A and B compartments were detectable [83]. This result is consistent with our gene analyses using previous parabolic flight datasets on chromosome cytobands, i.e., G-banding by Giemsa stain (Figure 5). 

However, the investigations of the overall intra- and interchromosomal interactions revealed interesting results (Figure 7b,c). In particular, small gene-rich chromosomes 16–22, with the exclusion of gene poor chromosomes 18 and 20, displayed increased intra- and interchromosomal interactions (Figure 7b). This effect is clearly significant for the comparison of hypergravity vs. 1g inflight but is even more pronounced for the comparison of microgravity vs. 1g inflight (Figure 7b,c). We observed a non-random overlap between the Hi-C-identified chromosomal interacting regions and the GRCRs from our gene expression analyses (*p*-value of 4.7 × 10^−5^ for hypergravity vs. 1gIF and 1.3 × 10^−9^ for microgravity vs. 1gIF; Figure 9). However, no overlap was identified for the gene poor chromosomes 18 and 20, which are located in the periphery close to the nuclear envelope [84].

We did not have a fully satisfying explanation on why this overlap was prominent for small chromosomes 16–22, but not for large chromosomes 1–15. However, microscopic techniques demonstrated that larger chromosomes are located closer to the nuclear periphery while smaller chromosomes are rather positioned in the center of the nucleus [85,86,87] (chromosomes 18 and 20 are an exception and are located rather at the periphery close to the nuclear envelope [84]). At the gene level, it has been shown that inactive genes and gene regions are often located in close vicinity to the nuclear lamina whereas active genes and gene regions are located in the center of the nucleus [88,89,90]. Goetze and colleagues observed similar results in fluorescent in situ hybridization (FISH) experiments. They showed that gene-dense regions called “ridges” are generally located closer to the nuclear center than gene-poor antiridges [60].

In addition to the abovementioned effects, we previously observed further ultrafast transcriptomic reactions and adaptations such as the ultrafast adaptation of the oxidative-burst reaction to microgravity. The comprehensive and rapid change in gene expression associated with regulatory RNAs and the stability of the cytoskeleton after long-term microgravity exposure suggest that the existence of rapid processes of gravitational force transfer into a cellular reaction [21,28,29,34,91].

Our current study demonstrated that rapid differential expression in chromosomal regions in response to gravitational changes, but not differentially expressed single genes, was highly conserved among different altered gravity comparisons. We further demonstrated that these findings were exclusively present in experiments under real altered gravity conditions, but not in control experiments and only partly in experiments under “simulated hyper- or microgravity”. These results were revealed by a GRCR correlation comparison analysis that was proven to be robust under various carefully designed parameter settings. We also found that the gene expression effects could not be described by functional gene set enrichment as a competing negative hypothesis. We proposed here that the underlying chromosome structure involved in the regulation of DGEs under true altered gravity was a mixed chromosome-length and chromosome-end model. This model could be improved by introducing parameters of Alu sequences and early vs. late replication domains. Additionally, TADs, known as regulatory elements, might be another key player of such regulation. 

The mechanical force transmission provides a possible explanation for these rapid changes. With the help of cytoskeletal elements anchored in the cell membrane, mechanical stimuli triggered by gravitational changes are perceived and transmitted into the cell interior within a few milliseconds and are projected into the nucleus [48,92]. The 3D organization of chromosomes plays an important role: a highly complex and ordered structure of CTs and subchromosomal structures exist in the nucleus. This determines gene regulation by chromatin topological changes and subsequently influences gene activation or repression which effects the transcription of genes [47,86,93,94]. CTs from large chromosomes have been described to be located at the nuclear periphery, while CTs from small chromosomes were rather found to be located close to the center of the nucleus [55]. Moreover, a nonrandom radial arrangement, as well as nonrandom neighborhood arrangements, has been identified for CTs. Based on the model, the transcriptionally active chromosome subregions are likely to be located centrally, while relatively inactive regions tend to be closer to the nuclear membrane, with chromosome telomeres pointing inwards and centromeres being closer to the lamina [54,60,86,95,96,97,98]. Additionally, gene-poor CTs (e.g., chromosome 18) and those with low overall transcriptional activity preferentially associate with the nuclear envelope, whereas gene-rich, highly transcribed, CTs (e.g., chromosome 19) are situated more towards the center of the nucleus [99,100,101]. This means a correlation of gene-dense chromosomes and gene-poor chromosomes with central and distal positions, respectively, could be assigned [102]. Furthermore, based on the high-resolution maps of chromatin, interaction units could be identified that mediate spatial gene expression control. Nuclear LADs are correlated with low gene expression and late replication timing [58,77,103,104]. TADs can be further divided into compartment A and compartment B, associated with early replication and active transcription and late replication and inactive genes, respectively [58,80,105,106,107,108]. Taking these previous findings and the rapid reaction times in our data into account, we propose a general model of rapid (or early) differential gene expression at the cellular level under altered gravity. The gravitational force is very small compared to Brownian motion within eukaryotic cells [109] and can probably only be detected with the help of the largest organelle, the cellular membrane [110], coupled to the mass of the cell which is organized and stabilized by the cytoskeleton.

Our results support the hypothesis that gravitational forces acting on the cellular membrane are rapidly and mechanically transduced by the cytoskeleton into the nucleus, moving CTs to new conformation states and their genes into more expressive or repressive environments. This could lead to territory-/region-specific DGE (Figure 9a) that was conserved between different experimental platforms and different directions of gravitational change (e.g., as a logical consequence, laminar regions can either stay or move inwards but never outwards, independent of direction of deformation). Following these rapid and transient effects, further gene-specific regulatory effects, such as transcription factors, may become the dominant influence in the expression patterns, leading to the seemingly uncorrelated behaviors of different altered gravity platforms on the single-gene level (Figure 9b) [31]. We therefore confirmed previous explanations on how gravitational forces influence early gene regulation in the cell, involving alterations in cell shape and mechanical stress [111] that propagates from the cellular membrane via the cytoskeleton to organelles including the nucleus [112,113]. We also proposed that the rapid and specific gene expression response to the altered gravitational force is encoded in the gene position within the chromosome architecture.

The existence of the co-expression of spatially co-localized genes was described already in 2001 by Caron and colleagues [114]. They reported the clustering of highly expressed genes to specific chromosomal regions which they called regions of increased gene expression (RIDGEs). A further characterization of these RIDGEs revealed that they are associated with chromosomes and chromosomal regions with high gene density but not likely with chromosomes known to have a low gene density such as chromosomes 18 and 21 [114]. Recent publications further identified that gene order in eukaryotic genomes is not random and genes with similar expression levels are clustered in the same genomic neighborhoods [115,116,117,118]. This phenomenon includes tissue-specific genes [119,120] as well as housekeeping genes [121,122] and is evolutionarily conserved [117,122,123,124,125].

It has been shown that upon altered gravity exposure, the nuclear morphology changes. These shape changes may result in the repositioning of the genome, as depicted in our mechanism model (Figure 9). The Hi-C data indicated that small gene-rich chromosomes located in the center of the nucleus are sensitive for these gravitational changes. The shape changes and relocation of the genome lead to DGE in GRCRs which overlap with intrachromosomal interaction sites. Furthermore, a major increase of intrachromosomal interactions sites was found in the comparison of microgravity vs. hypergravity. Additionally, interchromosomal interactions were enhanced with new interactions not only within the small chromosomes 16–22, but also between the small chromosomes and the large chromosome 3. We hypothesized that this increase in interaction depends on the microgravity condition where the cells and the nucleus are under reorganization upon sensing altered gravity and change in size and conformation. This conformational change leads to more space for certain parts of the genome (especially where the small chromosomes locate) to be more accessible, to unfold, therefore enabling enhanced intrachromosomal as well as interchromosomal interactions.

When considering how gravity-induced changes of the nuclear shape could promote chromatin reorganization, the interaction of the nuclear envelope with histones, especially histone H3, could be an important feature in gene expression regulation. Histones are frequently modified by post-translational modifications including methylation, acetylation, phosphorylation, and ubiquitylation to induce transcriptional regulation. These modifications influence the chromatin structure and represent binding sites for diverse transcription factors and are therefore important for gene expression, DNA repair, replication, chromatin compaction, and cell cycle control [126,127,128]. Among the histone post-translational modifications, H3K9 acetylation and trimethylation are hallmarks of transcriptional regulation and heterochromatin structure [129,130,131]. During histone acetylation, a negative charge is added to a lysine of the n-terminal end of histones. This negative charge causes the repulsion of the negatively charged DNA, inducing a relaxation of the chromatin structure. The opening of the chromatin allows then the binding of transcription factors and increases gene expression [131]. These findings were complemented with experiments investigating the influence of altered gravity on histone acetylation in human Jurkat T cells and human CD4+ T lymphocytes. During the suborbital ballistic rocket flight of the MASER 12 mission, we were able to detect a reduced acetylation of H3K9 in activated and non-activated human CD4+ T lymphocytes in altered gravity [21]. Furthermore, the histone acetyltransferase (HAT) inhibitor reduced p21 Waf1/Cip1 expression after 20s of microgravity [23]. In line with our results, Koaykul and colleagues found that simulated microgravity-coordinated cytoskeleton–lamin reorganization led to the suppression of histone modification in bone marrow-derived human mesenchymal stem cells [132]. Histones could therefore be a main connector of altered nuclear shape and gravity-induced DGE. Consequently, correlating histone modification and binding with gravity-induced DGE will be in the scope for future studies.

The context of the “exposome” concept addresses the combination of different stressors during a space flight. Thus, a comparison with other stressors rather than gravity should be considered. In this context, heat shock as a generic stress factor was an interesting candidate for an investigation on the GRCR occurrence and distribution. It yet remains unclear, if the mechanism hypothesized in this paper or the observed reactions is specific to altered gravity or if there are further factors that could cause such effects. In particular, the early heat shock response shows global dynamics that are comparable to some extent to the transcriptome-wide effects of short altered gravity exposure. Duarte and colleagues reported that drosophila cells show global effects of the nascent transcript pool after 20 min of heat shock treatment [133], and a similar behavior was reported for human K562 cells at 42 °C after 30 min of heat shock treatment [134]. Heat shock has been described to show a global effect on the transcriptome pool of mouse embryonic fibroblasts (MEFs) that affects around 3000 genes after 12 min of exposure and around 9000 genes after 60 min [135]. The fraction of DEGs is comparable to the results from the comparison TEXUS-51 microgravity versus 1gIF reference to some extent. Since the investigations on heat shock reactions were based on precision nuclear run-on sequencing (PRO-Seq), a direct GRCR analysis-based comparison as performed here was not possible. Interestingly, the number of DEGs in cells of the immune system seemed to be significantly lower after prolonged exposure to microgravity than directly after the onset: In space experiments after 1.5 h of microgravity, as well as in experiments after 4 h of simulated microgravity on the RPM, less than 100 DEGs were detected [15,16,136]. In contrast, other stressors resulted in significantly higher effects after prolonged exposure: For example, in human peripheral blood mononuclear cells (PBMCs) 552 genes were differentially experimented after 60 min post-exercise [137], and 1970 genes were differentially experimented after 18 h dexamethasone treatment [138]; 1 Gy neutron radiation resulted in up to 6226 DEGs in mouse blood 1–7 days after irradiation [139]. In contrast, tobacco smoke induced only 153 DEGs in human PBMCs after 5 min [140]. Thus, gravity changes seem to induce a much faster response and adaptation than other stressors, suggesting different underlying molecular processes.

Currently, the temporal dynamics and coherence of altered gravity response is not well understood, since most space and microgravity experiments cover only single or very few time points for operational reasons. On a single gene level, the overlap between the pools of DEGs after 20 s and after 5 min of microgravity is limited and follows an adaption scheme, where initially DEGs return to their base levels [31,34]. Recently, we found that also in hypergravity, all initially altered transcripts fully adapted after 15 min [63]. Thus, gravity changes seem to induce a much faster response and adaptation than other stressors, suggesting different underlying molecular processes.

These experiments were a model system in two respects: first, we used isolated cells without tissue and organ relation, and second, we did not use primary cells due to operational reasons during the flight missions. In the applied model system, the hydrostatic force acts in addition to the direct gravity effect in the 1 g experiments. Transferability to the in vivo situation of the organism is therefore only possible, where cells have no contact with a matrix structure such as connective tissue fibers or other cells. Thus, the model used here serves to identify direct gravity sensitivity by excluding or controlling indirect matrix-associated effects. Through this deliberately reductionist approach, empirical experimental conclusions about direct gravity effects in human cells become possible, and thus, fundamental biological conclusions. An integration in the totality of a complex cell system or an organism would require a completely different experimental setup. Therefore, our model system is limited to the identification of fundamental principles of gravity–structural–functional relations. According to the self-organized criticality (SOC) theory [141], a rapidly challenged gene-expression stability as a consequence of a structural response to microgravity could potentially drive the overall system into cell-fate options characterized by the expression of well-defined transcriptional programs [53]. The relative robustness of genome expression pattern across different gravity conditions has been demonstrated in tumor [142] and immune cells [31,33]. The rapid adaption of gene expression response [30], cytoskeletal organization [39], and cell function [28] and metabolism [26] to altered gravity environments underlines the systemic self-organized behavior of complex living systems [53,141], which is highly sensitive to modifications in the nonequilibrium dynamics, as we have demonstrated in our study. Thus, it is conceivable that highly dynamic rapid coupling effects between the gravity environment and 3D chromatin structure lead to the rapid response and adaptation of genome activity, which in turn induce robust longer-acting genomic and cellular changes in a nonequilibrated cellular state. 

Therefore, there may be a link between our findings of rapidly structural triggered and adapting effects and the observation of gene-expression networks supporting very different phenotypes by coordinated “profile preserving” modifications [53] in nonequilibrated systems in microgravity. Here, only coordinated and combined short-term and long-term experiments on suitable experimental platforms could bring an answer, a high technical and operational challenge that has not yet succeeded. The fast and coordinated whole-genomic response to changes of gravity could thus have fundamental effects on the whole reaction and adaptation situation of the cell in the interaction with its mechanobiological microenvironment, in the form of a general “field effect” of the gravitational field on biological processes. Understanding these “field effects” can only be conducted with a truly systemic and integrative approach.

## 4. Materials and Methods

### 4.1. Experiments in Altered Gravity

Transcriptomics samples were acquired from cell cultures of Jurkat T cell lines during the 23rd DLR PFC, the TEXUS-51 ballistic rocket campaign, and a GBF campaign based on Jurkat cell lines. Comparison experiments were acquired during another GBFs campaign based on the U937 cell line. An overview of all exploited experiments and sample groups is available in Table 1 and Figure 1.

During the parabolic flight, the following sample groups were investigated: Hardware 1× *g* ground control, 1× *g* onboard control, 1.8× *g* hypergravity (representing the baseline immediately before the first microgravity phase), and the microgravity phase (Table 1 sample nomenclature; Figure 1 showing the experiment fixation scheme). Right before the initiation of the first parabola, the 1× *g* board control sample was acquired. During the first parabola, the cells were consecutively exposed to 1.8× *g* and 0× *g* for 20 s and lysed at the end of each phase. 

On the suborbital flight of the TEXUS rocket, the cells experienced vibrations and hypergravity of up to 13.5× *g* for 75 s after lift-off. The corresponding cell samples were fixed directly at the end of this phase (t = 75 s after launch) and represent the baseline immediately before the microgravity phase. At the end of the subsequent microgravity phase (t = 375 s after launch), another sample set was fixed, so that the cells were exposed to microgravity for a total of 300 s. Additionally, the samples on an onboard centrifuge that supplied an onboard 1× *g* control environment during the microgravity phase were fixed in parallel to the microgravity samples (t = 375 s after launch). Further, there was a 1× *g* ground control group in which the cells were placed with inflight-identical hardware and fixed simultaneously to the microgravity samples. A cell culture control was also included in the experiment to identify hardware-related effects.

A third set of transcriptomics experiments was performed on Jurkat immune cell lines in our GBFs, which included a fast-rotating 2D pipette clinostat (60 rpm) and a pipette centrifuge with a maximum speed of 9× *g*. The cells were clinorotated at 60 rpm and centrifuged at 9× *g* at 37 °C for 300 s and fixated immediately thereafter. Then, the 1× *g* controls were placed at the base plate of the clinostat for the duration of the experiment to monitor hardware effects including instrument vibrations and fixated after 300 s.

Control experiment (referred to as control experiment 1) samples were from another ground-based campaign on U937 cells and did not correlate to altered gravity samples included in this study. Then, 1 g controls (sample group 1) were placed at the base plate of the clinostat for the duration of the experiment to monitor hardware effects including instrument vibrations and were fixated after 300 s. Further baseline samples (sample group 2), in which the cells were aspirated in a pipette and immediately released and fixed, were used to monitor the situation immediately before the vector-averaged gravity phase and to be able to exclude mechanical forces applied by pipetting the cell suspension up and down. Additionally, cell culture controls (sample group 3) that had no direct contact with the experimental hardware were directly taken out of the cell culture and lysed immediately.

An external reference control experiment (GEO access number: GSE98694; referred to as control experiment 2), performed by van Leeuwen-Kerkhoff et al. [62], included human 6-sulfo LacNac-positive cells (slan^+^: sample group 1) and two types of myeloid dendritic cells (CD1c^+^ norm: sample group 2, and CD141+ high: sample group 3), with all derived from donor blood samples buffy coats. These external samples were included in this study, since they represent immune-associated cells, and were analyzed on the same microarray as used in the aforementioned studies, which prevents technical noise effects due to differing analysis platforms.

### 4.2. Generation of Transcriptomics Data

**Figure 10:**
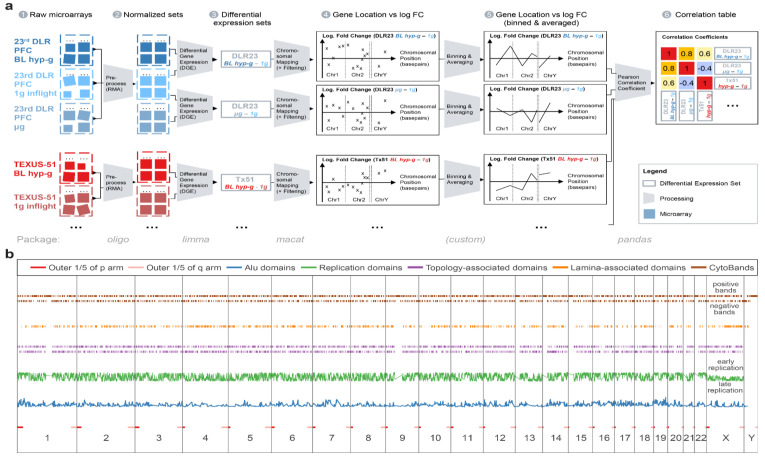
Components of the analysis pipelines. (**a**) Schematic overview of the sample analysis pipeline. (1) Raw array data were available as CEL files for all compared experiments (except for control experiment 2 that was already in a normalized state). (2) Raw array data were RMA-normalized (Robust Multiarray Averaging) and converted into experiment-wise transcript cluster data. (3) Differential gene expression comparisons were calculated with Bioconductor limma by fitting a linear Bayesian model to the data. (4) Data were annotated with their distinct localization on the human genome and mapped to all chromosomes. Only uniquely mapping genes were kept; additionally, only the top 2000 differentially expressed genes were included. (5) Genes were binned into bins of 10,000,000 bp and averaged within bins. The bin logarithmic fold change averages were plotted at the middle of the bins, chromosome by chromosome. (6) The Spearman correlation coefficients of all bin averages were calculated between different DGEs comparisons, yielding a correlation coefficients table with all DGEs comparisons. The value between two identical sets is always 1 and is represented on the diagonal. (**b**) Distribution of all DNA motifs that were used in linear models for the hypotheses testing. The horizontal axis shows the chromosomes, from chr1 to chrY, aligned from the beginning of the p arm to the end of the q arm. The outer 1/5 of the p/q arm are displayed in red, and the number of Alu domains within 100,000 bp on the same chromosome is displayed per location in blue, on a relative scale.

Gene expression profiling was performed on Affymetrix HTA2.0 chips (Affymetrix Ltd., Santa Clara, USA), covering 44,699 protein-coding transcripts and 22,829 nonprotein-coding transcripts. Microarray hybridization and readout were performed externally at the Core Facility Genomics of the Medial Faculty Muenster (Muenster, Germany). The processing of raw. CEL files was performed consecutively by the open source Bioconductor packages [143], following the principle of the feature-level extraction output (FLEO). Probe set data (Figure 10) were aggregated and averaged experiment-wise by robust multiarray averaging using the oligo 1.48.0 package (Figure 10 (a2)). The normalized and aggregated Affymetrix HTA2.0 chip dataset GSE98694 was pulled from the GEO database. Data quality was verified with the help of the arrayQualityMetrics 3.40.0 package. Batch effects were detected for the 23rd DLR parabolic flight dataset on the level of two different microarray hybridization and readout dates, which has been identified by principal component analysis as a main source of technical noise. 

The normalized transcript cluster datasets were annotated by using the open source hta20transcriptcluster.db 8.7.0 package. Transcript clusters were summarized to gene-level data. DGE was calculated by fitting a linear Bayesian model with the help of the limma 3.40.6 package (Figure 10 (a3)), which resulted in a list of logarithmic fold changes (logFC) per gene. For the DLR23 dataset, batch effect correction was performed precedingly by limma to remove batch effects as described above. 

For the label permutation analysis, the same protocol was applied, but the data were intentionally labeled incorrectly, so that a group of microarrays always contained three samples that were exposed to the mentioned condition and three to opposite one so that the true effect should disappear upon comparison and only technical noise (and spread within the sample groups) should remain. All pairwise combinations of label permutations were calculated.

### 4.3. Chromosomal Mapping

Chromosome mapping analysis was performed using annotations from the biomart package. Genes were annotated with their respective positions on the genome by their ENTREZ ID and mapped on the Genome Reference Consortium hg38 human reference genome. All unique genes with a single locus were plotted chromosome by chromosome with the location on the respective chromosome with one chromosome following the other on the *X* axis and the logarithmic fold change of the DGE condition on the *Y* axis (Figure 10 (a4)). 

### 4.4. Spearman Correlation Coefficients

To provide a comparison between different datasets with inconsistent overall intensity of fold changes between different groups, instead of a fixed logFC/*p* value cutoff, a relative cutoff for DGE was chosen: The data were filtered by keeping the top 2000 differentially regulated genes (absolute value of logFC). Following, the data were binned and averaged in equal distances of 5,000,000 base pairs with a binning window size of 10,000,000, leading to a 2× oversampling which prevented effects by an arbitrarily chosen binning window border. These binned data were plotted by placing the location of the middle of the binning window (in base pairs) chromosome by chromosome as a location value and by placing the average logarithmic fold change, weighed by the average expression value per gene, on the vertical axis (Figure 10 (a5)). The Spearman correlation coefficient (rank coefficient with a minimal number of assumptions) of all combinations of binned data traces was calculated with pandas’ 0.25.3 corr function, grouped and plotted as a clustered 2D heatmap (Figure 10 (a6)). The Spearman correlation coefficient can take values of −1 (perfect anticorrelation), 0 (no correlation), and 1 (perfect correlation). For each coefficient, the FDR-corrected *p*-value was calculated to test the statistical significance of the correlation between the binning averages of two comparisons.

For the altered parameter analysis and the structure width analysis, one or two parameters were altered as described in the corresponding figures, and all other parameters were set as described above. The analysis was performed as described. For the label permutation analysis, the average of all pairwise combinations per comparison was calculated.

### 4.5. Shuffling Differential Gene Sets—Expected Average Correlation Coefficients 

The location values of the annotated expression sets were randomly shuffled before filtering, without separating same genes from different datasets, keeping pairs together. The aforementioned filtering, binning, and averaging pipeline were then applied to the shuffled datasets, and correlation coefficients were calculated. This procedure was repeated 1000 times to bootstrap the average and standard deviation of the resulting correlation coefficient distribution per condition comparison. Quantification of difference in correlation was performed by comparing the Spearman correlation coefficient before and with the mean value after shuffling in absolute units of correlation coefficient and in multiples of the standard deviation of the coefficients after shuffling. For each coefficient, the FDR-corrected *p*-value was calculated to test the statistical significance of the correlation between the binning averages of two comparisons.

For the altered parameter analysis and the structure width analysis, one or two parameters were altered as described in the corresponding figures, and all parameters were set as described above. The analysis was performed as described. For the label permutation analysis, the average of all pairwise combinations per comparison was calculated.

### 4.6. Model Simulation

Two datasets were simulated. First, a random noisy background was mapped to all uniquely mapping genes by drawing random variables from a β-distribution with parameters a = 1 and b = 1 centered at 0 and with a maximum logarithmic fold change of ±0.5. The noisy values between the two simulated datasets were independent of each other and results in a Spearman correlation coefficient of 0 on average.

For the “strong correlated genes” model, between 0 and 1000 genes were randomly selected out of all genes present. These got assigned a logFC between ±1 and ±10 with a maximum difference of ±0.1 between the two simulated datasets, resulting in a set with firstly a random, uncorrelated background of genes with a weak logFC and secondly pairs of strongly DEGs that were randomly distributed over the genome. 

For the “responsive chromosomal regions” model, between 0 and 500 genes were selected as the center of GRCRs. For each site, genes within a neighboring distance got assigned logarithmic fold changes between ±1 and ±10 with the same sign for all genes within one site. The maximum neighboring distance was randomly defined per site as between 500,000 and 1,500,000 base pairs, leading to region site sizes between one and three million. Between the two simulated datasets, only the location, the size of the locus, and the direction of the fold change (the sign) were defined, and indirect correlation between gene pairs was defined.

The analysis pipeline that was applied on the real data was also applied on the simulated datasets. Binning, filtering, and averaging were performed on the data, and a Spearman correlation coefficient was then calculated between the two simulated sets. A pairwise shuffle bootstrapping of genes was then performed before calculating the expected average correlation coefficient. This analysis was bootstrapped 100 times per simulated scenario with a distinct number of genes with a strong correlation/number of sites with GRCRs. The average values of the Spearman correlation coefficient for different numbers of genes with strong correlation/colocalizing reactive chromosomal regions were calculated and displayed in a diagram, and a moving average line with a window size of 30 was added.

### 4.7. Gene Set Enrichment Analysis

GSEA pathways were pulled from the msigdb database with the help of the msigdbr 7.0.1 Bioconductor package. Mean signed deviation values were calculated from the DGE list results from the *limma* package and used as ranking variable. GSEA enrichment scores were calculated with the help of the *fgsea* 1.10.1 (fast gene set enrichment analysis) package. For the benchmarking between DGE sets, the correlation coefficient for the normalized enrichment scores of all msigdb gene sets was calculated. Additionally, after filtering for gene ontology (GO) sets, the number of shared sets within the top 15 upregulated and top 15 downregulated GO sets was identified. Accordingly, the number of shared sets within the list of differentially expressed GO sets (defined as enrichment *p*-value below 0.05) was calculated. The DGE sets with the strongest and the weakest influences of the shared gene sets were identified per analysis parameter. The Spearman correlation coefficient for each comparison was calculated, based on the relative difference in correlation (in multiples of standard deviation) and benchmarking parameter. The same GSEA analysis was conducted again only considering genes that were shared between two DEG sets in their top 2000 genes.

### 4.8. Hypothesis Feature Annotation

Each gene in each DGE set was annotated with feature ids for all tested hypotheses. The fold change differential expression data were tested for potential correlations with locations on different chromosomes, location within the outer 1/5 of the p/q arms of chromosomes, density of Alu repeat domains, early vs. late replication domains, chromosome cytobands, TADs, and LADs. Chromosome p/q arm locations were extracted from the UCSC chromosome information track, Alu repeat locations form the UCSC RepeatMasker for hg38 track and then filtered for Alu elements, replication timing data from the Replication domain GM12878 (Int90901931) dataset (human lymphocytes), cytobands from the UCSC Cytobands hg38 track, TAD locations from the ENCODE platform for IMR90 cells for hg19 and then converted to hg38 annotation by the UCSC uplift tool (successful conversion of 3055 records and failures of 64 records), and LAD locations from UCSC track NKI LAD for hg19 and then converted to hg38 annotation by the UCSC uplift tool (successful conversion of 1296 records and failures of 6 records) for human fibroblasts (conservation rate up to 95%).

Alu sequence density was assessed by counting the number of Alu sequences within a distance of ±50,000 bp. LAD association was defined by genes that were located within an LAD or neighboring a domain by a maximum of 10,000 bp. 

A map of all annotated features used in this analysis was created (Figure 10b). 

### 4.9. Subsetting Correlation Coefficient Analysis

The Spearman correlation coefficient analysis was performed on DGE sets that were separated into two subsets based on different hypothesis features. The sets were split into genes that were located on long chromosomes (chr1–chr12, chrX) and short chromosomes (chr13–22, chrY), into subsets of genes on the outer 1/5 of the p and the q arm or the rest of the chromosomes, into subsets of genes in areas rich in Alu repeats (>50 Alu repeats within 100,000 bp) and poor areas (50 or less Alu repeats within 100,000 bp), into subsets of genes that replicated early during cell division (replication index: >0) and replicate late (replication index: <0), and into genes on positive Giemsa stain cytobands (pooling gpos25, gpos 50, gpos 75, gpos100, and gvar) and on negative Giemsa stain bands (gneg). For TADs and LADs, around 85% of the top 2000 DEGs were located on TADs and around 20% of the top 2000 DEGs were located on LADs, which did not support a sub-setting into groups of approximately the same size.

These subset datasets were handed over to the same binning, averaging, and correlating pipeline as previously described. Correlation coefficients before and after shuffling were acquired for each subset for the TEXUS-51 and the 23rd DLR PFC datasets, and the number of contributing bins was reported not to overestimate findings based on very small numbers of bins. For chromosome ends, Alu repeats-rich/poor regions, Cytobands, and replication domains, the minimum number of genes per bin was set to five to account for the smaller density of genes per subset. For LADs and TADs, no parameters could be found that facilitate an evaluation based on reasonable numbers of bins for both the subsets within and outside of these features, which is a consequence of the very unequal distribution of regions inside and outside such features.

### 4.10. Linear Modeling Analysis

Simple linear models based on each hypothesis were created and fit on 90% of the differential expression data, measuring the predictive strength on the remaining 10% of data. The R^2^ value as an indicator of predictive strengths was calculated for a predictive model based on the fold changes of the top 2000 DEGs (corresponding to the GRCR analysis), the top 5000 DEGs (extended set potentially containing more signals but also more noise/unaltered genes; harder to predict), and all 16,800 uniquely mapping genes (containing a lot of noise; hardest to predict). This approach was bootstrapped 1000 times with different random 90:10 splits of the data and averaged to yield the average coefficient of determination $\bar{R^{2}}$.

Technically, this was performed using an ElasticNet optimizer, penalizing diverging parameters with a combined l1 and l2 norm. The calculations were performed with the open source python package scikit learn 0.22 [144]. The predicted variables ($Y_{i}$) were set as the fold changes of the top 2000/top 5000 or all 16,800 DEGs. The independent variables were set based on the tested genome structure. To be able to assess the true predictive strength value, the dataset was randomly split into modeling data (90%) and test data (10%). One thousand iterations of different splits were calculated to provide the independence of the split. The $R^{2}$ values of the fits were averaged; the standard error of the mean was below 0.001 for all reported values. For the two combined models including more than one feature, quadratic interaction terms were also included. For the color coding of R^2^ values, the highest predictive strength per set of the predicted variables was identified and set to dark red, 0 or less predictive strength was set to white, and all values were colored in different shades of red.

The different models were shown as follows.

#### 4.10.1. Chromosome Lengths

$$Y_{i}=\sum_{j}s_{\mathrm{ij}}{\;w}_{j}+e_{i},$$

where $j\in \left\{1,\;\ldots ,\;22,\;X,Y\right\}$ are chromosomes, $s_{\mathrm{ij}}$ is the chromosome index (1 when j is the chromosome of gene i, 0 otherwise), and $w_{j}$ is the chromosome-specific coefficient. Therefore, this model has 24 independent variables.V.

#### 4.10.2. Chromosome Ends

$$Y_{i}=\sum_{j}s_{\mathrm{ij}}{\;w}_{j}+e_{i},$$

where $j\in \left\{p,\;q,\;0\right\}$ is the 1/5 outer ends of p/q arms of chromosomes or 0 as not inside p or q, $s_{\mathrm{ij}}$ is the region index (1 when gene i is in a region indicated by j, 0 otherwise), and $w_{j}$ is the region-specific coefficient. Therefore, this model has 3 independent variables.

#### 4.10.3. Alu

$$Y_{i}=s_{i}\;w+e_{i},$$

where $s_{i}$ is the gene-specific Alu-domain counter (number of Alu domains within ±100k bp on the same chromosome) and w is the Alu-domain regulation coefficient. The number of coefficients n is 1.

#### 4.10.4. Replication Domains

$$Y_{i}=s_{i}\;w\;+e_{i},$$

where $s_{i}$ is the replication domain-specific timing number (timepoint of replication, from −1 as a late replication to 1 as an early replication) and w is the replication domain regulation coefficient. The number of coefficients n is 1.

#### 4.10.5. LADs

$$Y_{i}=\sum_{j}s_{\mathrm{ij}}{\;w}_{j}+e_{i},$$

where $j\in \left\{\mathrm{no}\_\mathrm{LAD},\;0,\;1,\;2,\ldots ,n\right\}$ is the index per LAD, $s_{\mathrm{ij}}$ is the LAD domain index (1 when gene i is in in a LAD domain indicated by j, 0 otherwise), and $w_{j}$ is the domian-specific coefficient. The number of coefficients n≈200 is for the top 2000 genes, the number of coefficients n≈450 is for the top 5000 genes, and the number of coefficients n≈920 is for 16,800 genes with slight variations between different sets.

#### 4.10.6. TADs

$$Y_{i}=\sum_{j}s_{\mathrm{ij}}{\;w}_{j}+e_{i},$$

where $j\in \left\{\mathrm{no}\_\mathrm{TAD},\;0,\;1,\;2,\ldots ,n\right\}$ is the index per TAD, $s_{\mathrm{ij}}$ is the TAD domain index (1 when gene i is in in a TAD domain indicated by j, 0 otherwise), and $w_{j}$ is the domain-specific coefficient. The number of coefficients n≈930 is for the top 2000 genes, the number of coefficients n≈1500 is for the top 5000 genes, and the number of coefficients n≈2090 is for 16,800 genes with slight variations between different sets.

#### 4.10.7. CytoBands

$$Y_{i}=\sum_{j}s_{\mathrm{ij}}{\;w}_{j}+e_{i},$$

where $j\in \left\{1,\;\ldots ,861\right\}$ is the index per cytoband from the giemsa stain of the human chromosomes at the highest resolution, $s_{\mathrm{ij}}$ is the cytoband domain index (1 when gene i is in in a cytoband indicated by j, 0 otherwise), and $w_{j}$ is the band-specific coefficient. The number of the used coefficients n≈230 is for the top 2000 genes, the number of coefficients n≈250 is for the top 5000 genes, and the number of coefficients n=255 is for 16,800 genes with slight variations between different sets.

#### 4.10.8. Chromosome lengths/ends

$$Y_{i}={\left(\sum_{j}s_{\mathrm{ij}}{\;w}_{j}+\sum_{k}s_{\mathrm{ik}}{\;w}_{k}\right)}^{2}+e_{i},$$

where $j\in \left\{1,\;\ldots ,\;22,\;X,Y\right\}$ are chromosomes, $k\in \left\{p,\;q,\;0\right\}$ are 1/5 outer ends of p/q arms of chromosomes or 0 as not inside p or q, $s_{\mathrm{ij}}$ is the chromosome index (1 when j is the chromosome of gene i, 0 otherwise), $s_{\mathrm{ik}}$ is the region index (1 when gene i is in a region indicated by j, 0 otherwise), $w_{j}$ is the chromosome-specific coefficient, and $w_{k}$ is the region-specific coefficient. Quadratic terms are included. Therefore, this model has 406 independent variables.

#### 4.10.9. Chromosome Lengths/Ends/Alu/Rep

$$Y_{i}={\left(\sum_{j}s_{\mathrm{ij}}{\;w}_{j}+\sum_{k}s_{\mathrm{ik}}{\;w}_{k}+s_{\mathrm{Alu},\;i}{\;w}_{\mathrm{Alu}}+s_{\mathrm{Rep},\;i}{\;w}_{\mathrm{Rep}}\;\right)}^{2}+e_{i},$$

where $j\in \left\{1,\;\ldots ,\;22,\;X,Y\right\}$ are chromosomes, $k\in \left\{p,\;q,\;0\right\}$ are 1/5 outer ends of p/q arms of chromosomes or 0 as not inside p or q, $s_{\mathrm{ij}}$ as chromosome index (1 when j is the chromosome of gene i, 0 otherwise), $s_{\mathrm{ik}}$ is the region index (1 when gene i is in a region indicated by j, 0 otherwise), $s_{\mathrm{Alu},i}$ is the gene-specific Alu-domain counter (number of Alu domains within ±100k bp on the same chromosome),${\;s}_{\mathrm{Rep},i}$ is the replication domain-specific timing number (timepoint of replication, from −1 as a late replication to 1 as an early replication), $w_{j}$ is the chromosome-specific coefficient, $w_{k}$ is the region-specific coefficient, $w_{\mathrm{Alu}}$ is the Alu-domain regulation coefficient, and $w_{\mathrm{Rep}}$ is the replication-domain regulation coefficient. Quadratic terms are included. Therefore, this model has 465 independent variables.

### 4.11. Pinpointing of DGE under Altered Gravity to Chromosomes

DEGs were identified out of all 16,800 uniquely mapping genes by filtering for *p* < 0.05 (uncorrected, to achieve better comparability between very different DGEs). DEGs were categorized by upregulated (logarithmic fold change > 0) and downregulated (logarithmic fold change < 0) genes. The percentage of differentially (up/down) regulated genes was calculated by diving the number of regulated genes by the total number of uniquely mapping genes per chromosome. Color coding was set to blue for real altered gravity and red for control samples in the main figure.

The modeling parameter $w_{j}$ for each chromosome within the “chromosome length” linear model was extracted from the ElasticNet linear model fit, as previously described from the average model from 1000 bootstrapped splits into the modeling and test data. The data were plotted chromosome by chromosome for different DGEs for the linear model for the top 2000 genes, the top 5000 genes, and all the 16,800 uniquely mapping genes. Real altered gravity conditions were compared to control experiment 2, and a second figure only including controls was added to Supplementary Materials. Chromosomes 18 and 19 were highlighted as reference points.

### 4.12. Hi-C Sample Acquisition

Hi-C samples were generated during the 4th Swiss PFC onboard the A310 ZERO-G from Novespace at Air Base Dübendorf, Switzerland on 11 June 2020. The sample acquisition conditions between the 23rd DLR PFC and the 4th Swiss PFC were comparable (highly standardized ground procedures, similar flight profile and identical sample points, identical aircraft, same cell line, and sources of consumables). The experiment consisted of four conditions with three samples per condition: hardware 1× *g* ground control, 1 g onboard control, 1.8× *g* hypergravity (representing the baseline immediately before the first microgravity phase), and the microgravity phase (same scheme as for 23rd DLR PFC, comparing the sample nomenclature (Table 1) and experiment fixation scheme (Figure 1)). Right before the initiation of the first parabola, the 1× *g* board control sample was acquired. During the first parabola, the cells were consecutively exposed to 1.8× *g* and 0× *g* for 20 s and fixated at the end of each phase. Fixation was performed by injecting an excess of formaldehyde in a PBS solution with a target concentration of 3%. After 3 min of incubation, excess 2.3 M glycine was injected into the sample. All steps were performed at 36.5 °C. After landing after 2 h experiment, containers were disassembled, and the samples were stored at 4 °C. The sample solutions were spun down, pellets washed, concentrated and stored at −150 °C. The Hi-C sample generation was performed by using the commercial ArimaKit from Arima Genomics (San Diego, CA, USA). Sequencing was performed by Arima Genomic (San Diego, CA, USA) with a minimum read number of 400 M per direction with a read length of 150 bp and a stranded paired end setting.

### 4.13. Hi-C Data Processing

Raw reads were processed by the Juicer Hi-C pipeline [145] pipeline and aligned against hg38. Data quality was assessed based on inter.txt quality report files from Juicer, which are available upon request. The aggregation of conditions, A/B calling, and interaction map plotting was performed with HiCExplorer [146]. A/B tracks where the orientation did not match the other samples were manually inverted. Differential Hi-C interaction calling was performed with the DiffHiC package [147], based on aligned reads (hg38) from the HiCUP pipeline [148]. Bin pairs with a size of 1 Mb were tested for significant interaction. The list of bin pairs was split into interchromosomal and intrachromosomal pairs. The latter was independently filtered for strong signals (normalized interaction signal > 0) to yield sufficiently strong bins. An FDR-corrected *p*-value cutoff of 0.25 (hypg vs. 1gIF) and 0.1 (µg vs. 1gIF) was chosen as the lowest FDR level. For interchromosomal bin pairs, significant differences were defined by an FDR cutoff of 0.33 (hypg vs. 1gIF) and 0.2 (µg vs. 1gIF), the lowest FDR level. For the overlap analysis, gravity-responsive transcriptional regions were defined by containing bin averages for both 23rd DLR PFC comparisons for the correlation coefficient analysis. Fisher’s exact test was performed on a contingency table with Hi-C bins in target regions vs. Hi-C bins which were not in target regions, against all possible bins within regions vs. all possible bins outside.

## Figures and Tables

**Table 1:** Overview of sample nomenclature for microgravity platforms and GBFs applied in different experimental research studies. Datasets generated by our experiments are “internal”, and publicly available datasets are “external”. Gravity conditions and times are given for parabolic flights, sounding rockets, and GBF experiments. GBF, ground-based facility; PFC, parabolic flight campaign; TX, TEXUS mission; Exp, Experiment; 1CCC, cell culture control; H/W, hardware; IF, in-flight; BL, baseline; hyp-g, hypergravity; μg, microgravity; n/a, not applicable.

	Experiment	Microarray Transcriptomics (Internal)				Chromosome Conformation Capture (Hi-C, Internal)		Microarray Transcriptomics (External)
	Study	23rd DLR PFC	TEXUS-51	GBFs Jurkat	Control Exp. 1 (GBFs U937)	4th Swiss PFC Hi-C		Control Exp. 2 (GSE98694)
**Gravity condition**	CCC 1× *g* ground	n/a	+	n/a	Sample Group 3	n/a	**Sample condition**	Sample Group 1 (slan+: sample group 1)
	H/W 1× *g* GC	+	+	n/a	n/a	+		Sample Group 2 (CD1c+ norm)
	1× *g* IF	+	+ (on board centrifuge, 300 s)	n/a		+		Sample Group 3 (CD141+ high)
	1× *g* GBF	n/a	n/a	1× *g* GBF (300 s)	Sample Group 1 (1× *g* GBF (300 s))	n/a		
	BL (directly before μg phase)	BL PFC hyp-g (1.8× *g*, 20 s)	BL-TX hyp-g (max. 12.6× *g*, 75 s)	n/a	Sample Group 2 (BL-GBF 1× *g*, approx. 10 s)	hypg: BL PFC hyp-g (1.8× *g*, 20 s)		
	Microgravity (μg)	μg (20 s)	μg (300 s)	sim μg (300 s clinorotation)		µg (20 s)		
	Hypergravity (centrifuge)	n/a	n/a	9 g (300 s centrifugation)		n/a		

## Data Availability

The datasets generated during and/or analyzed during the current study are available in the GEO (Gene Expression Omnibus) repository (www.ncbi.nlm.nih.gov/projects/geo; accession no. GSE94256, GSE101309, GSE101102, and GSE174291; accessed on 1 July 2021).

## Supplementary Materials

The following are available online at https://www.mdpi.com/article/10.3390/ijms22179426/s1.
