# Supplementary material for: Gravitational Force—Induced 3D Chromosomal Conformational Changes Are Associated with Rapid Transcriptional Response in Human T Cells
Source: Int J Mol Sci. 2021 Aug 30;22(17):9426. doi: 10.3390/ijms22179426 (PMC8430767; doi:10.3390/ijms22179426)
Supplement: Supplementary file 1 [file ijms-22-09426-s001.zip › supplement_rev.pdf]

Supplementary Text 1. GRCR Analysis.

S1.1 Quantification of gravity-responsive chromosomal region colocalization

The complex structure of gene distribution and coupling of fold changes among adjacent genes, for which the type of model heavily influences outcomes [1], renders it almost impossible to determine if a given distribution of differentially expressed genes (DEGs) by itself is non-random (Supplementary Figure S5a). Additionally, the utilized microarray (Affymetrix Human Transcriptome Array 2.0) does not cover all genes but only a subset, which complicates distribution statistics. Therefore, the question was whether the given distributions for DEGs in altered gravity comparisons exclusively share GRCRs at the same locations in contrast to control data sets. Control data sets could still bear structures with local aggregation that would classify as GRCR. Therefore, adequate differentiation between the control data sets and samples that were exposed to altered gravity is necessary. To address this question, we quantified the distribution of gravity-responsive chromosomal regions identified by one-dimensional mapping among all differential gene expression comparisons to assess whether there are any conservative regions (or consistent patterns) among these comparison data sets.

Firstly, the data sets were filtered for genes that do not show significant differential expression to eliminate the background. The data set behave very different in this regard: We observed different expression levels with different p values between gravity-related comparisons, likely due to different exposure times and effect strengths: for example, even if the expression of a gene came to complete halt in altered gravity, the RNA concentration and therefore the fold change had significantly less time to change during a parabola of 20 seconds compared to a sounding rocket flight of 5 minutes. However, the aim was to compare the distribution of gravity-responsive chromosomal regions among experiments. Whether these regions show the exact same strength of fold changes (FC) at identical p values was not important for this aim. Therefore, no fixed FC or p value cutoff was set, but a dynamic filter setting based on limma linear modeling was applied. The top 2000 DEGs based on absolute fold changes were used for subsequent comparisons, which ensured maximum comparability. This approach provides a reasonable average p value below 0.05 for all data sets (comp. Supplementary Table S2).

Secondly, these 2000 DEGs from each comparison were binned based on their locations and averaged, using windows of 10 million base pairs (bps) over chromosomes, to be able to compare effects on the level of sections, not on single genes. Most importantly, this would also allow comparability if GRCR were composed of different genes from the same section that exhibited the same regulation. The size of 10 Mbps was chosen in accordance with the observed sizes of such regions (compare Figure 2) and for topologically associating domains (TADs) as well as other known chromosomal structures to comfortably fit in. TAD is a recently discovered genomic structure with a median size of 1.15 Mb and maximum up to 3 Mb for human cells [2]. To prevent cutting GRCRs apart by arbitrarily chosen binning window borders, a two-fold oversampling with 50% overlap was performed (Supplementary Figure S5b, details in materials and methods). Only bins with a minimum of 10 genes were included to limit the influence of single genes in sparse regions which do not qualify as gravity-responsive chromosomal regions. This data processing resulted in a bin average vector, carrying the bin averages or NAs if a bin did not meet the minimum number of genes criteria. The parameters of this analysis were carefully chosen based on these considerations. However, there is always the possibility that findings are random and only appear for one specific set of parameters. To mitigate this risk, the analysis was also conducted with altered bin sizes, altered minimum number of genes, and altered number of top DEGs. This stability analysis should change the absolute results values but not the relative differences between comparisons. See chapter "The analysis results are robust against parameter variations and not caused by technical noise".

Thirdly, we tested whether the distribution of gravity-responsive chromosomal regions is conserved between different comparisons. It was tested if this conservation significantly differs from the null hypothesis: a non-significant average permutation of the randomized data set, represented by the average expected correlation. The Spearman correlation coefficients (cc) and corresponding p values from the Spearman test between the bin average vectors of two comparisons were calculated, first on the average expected data set, then on the actual observed data set. The average expected correlation coefficients and FDR-adjusted p values have been calculated by bootstrapping 1000 permutations of the fold changes of

all genes within the genome before filtering and binning, without separating identical genes between comparisons. Therefore, identical genes in different comparisons still colocalize but are separated from their neighboring genes. Again, due to the dependency on models of fold change distribution [1], this did not necessarily reflect the true natural state but served as measure to understand whether the observed distribution significantly differs from a random distribution. A highly increased correlation coefficient with significant p value for the actual data set compared to the average expected data set indicates that the distribution of genes in gravity-responsive chromosomal regions is non-random.

The resulting correlation clustermap of average expected distributions (Supplementary Figure 5c) showed a significant signal for all intra-experiment correlations of comparisons. The highest levels were achieved for control experiments and the simulated gravity experiments. For all inter-experiment correlations however, the values were below 0.2 with non-significant p values. Consequently, a certain intra-experiment correlation is expected simply by the pure distribution of fold changes. These were unaltered by randomization, in contrast to specific structures which would average out by randomization. Therefore, the observed intra-experimental signal observed in Supplementary Figure 5c cannot derived from gravity-responsive chromosomal regions.

The actual Spearman values (Supplementary Fig 5d) on the other hand showed again highly significant intra-experiment correlations, interestingly with increased levels. Additionally, correlation was significant also for some inter-experiment correlations: between real altered gravity experiments, partly between GBFs and TEXUS-51, and partly anti-correlating between TEXUS-51 and control experiment 2. The strong actual correlations between the two hypergravity resp. the two microgravity experiments showed comparable behavior to intra-experiment comparisons. On the other hand, the inter-experiment correlation coefficients were below 0.2 with non-significant p values for the average expected distributions. This indicates a conservation between gravity-responsive chromosomal regions with differential gene expression patterns that were not driven by random distribution. These gravity-responsive chromosomal regions do not necessarily have to be driven by the same genes in two correlating comparisons, but also by different, neighboring genes.

Following, selected comparison vs comparison correlations were highlighted, including gravity-related intra/inter-experiment comparisons, simulated altered gravity comparisons and control experiments (Figure 3). Correlations were regarded in absolute (difference in correlation coefficient) and relative numbers (multiples of standard deviation of the expected correlation coefficients $n \cdot \sigma$). The smaller the n, the more likely the actual and the average distribution were part of the same statistical distribution, therefore not significantly different. If differences in absolute values and in multiples of n were large, the two comparisons likely had overlapping GRCRs.

S1.2 The analysis results were robust against parameter variations and not caused by technical noise

To test against any potential bias by the settings of filtering, binning and averaging parameters, a stability analysis was performed: Further analyses were conducted under altered analysis parameters (see Supplementary Figure 6). These altered parameters include: A. The relative filtering for the top 2000 differentially expressed genes was removed and all 16800 uniquely mapping genes included (S6a); B. The filter for minimum numbers of genes per bin was removed (S6b); C. The binning window distance set was changed to 10 million bp, omitting oversampling, (S6c); and D. The bin size set to 1 million bp whilst setting the minimum number of genes per bin to 1 (S6d). Every comparison showed the same relative result, some with reduced effect strength. A more detailed analysis of the effects of different bin sizes from 1 million bp to 20 million bp was performed (Figure S7). The relative order of correlation differences remained the same for any chosen bin sizes, but the contrast in correlation was smaller for smaller bin sizes with a maximum at approximately 8-10 million. Therefore, the results were robust against parameter variations and not biased by chosen analysis parameters.

To assess whether the observed effects were influenced by other biological and technical factors such as distribution of microarray probes, independent of the conducted experiments, a label permutation analysis was performed (Supplementary Figure S8). Briefly, differential gene expression was re-analyzed for all data sets with permutated sample group labels. Thereby, sample groups always consisted of 50% of samples that have previously been in the group and 50% of samples from the group they were compared to, to statistically eliminate the true effect. In reality, due to differences in samples and

microarrays, a remaining residual effect will still lead to some correlation. This analysis was performed pairwise for all possible permutations. Averages of the correlation coefficients before and after shuffling were calculated with standard deviations and standard errors of means. The results were no longer significant for any comparison group, and the standard deviations overlapped for all groups. Consequently, the previously observed loss in correlation strength effect after shuffling was not caused by technical noise or by the pure arrangement of genes on the chromosomes without any external stimuli but was depending on real altered gravity.

S1.3 An exemplary fine-structure analysis revealed structural differences in regions with strong and with weak increase in correlation strength

To have a better understanding of underlying factors that drive the different behavior of comparisons, a chromosomal region was selected that showed strong differences between actual vs expected correlation strength for one comparison and only weak differences for another comparison. Chromosome 2 showed a correlation strength difference of 0.38 for the two microgravity comparisons and of only 0.03 for the two GBF comparisons (Supplementary Figure 9). The mean absolute differences in logFC between all genes on chromosome 2 resp. the first 50 million bp of chromosome 2 fluctuated a lot, but was constantly higher for the microgravity comparison (Supplementary Figure 9b). In the light that correlations that showed a strong difference between expectation and actual Spearman correlation coefficients shared less genes in their top 2000 genes, this finding was coherent with a model where colocalizing gravity-responsive chromosomal regions were driven by different genes in different comparisons. The distribution of all fold changes on the first 50M bp of chromosome 2 for all four comparisons showed more strongly regulated gene pairs for the GBF comparisons (Supplementary Figure 9c/d) which became more prominent after filtering for the top 2000 genes (Supplementary Figure 9e/f): The GBF comparisons shared considerably more genes within the top 2000 genes (12 withing the first 50M bp, 75 on the entire chromosome) whereas the microgravity correlation had less (5 resp. 20). This example illustrates the effect of shared genes within the top 2000 genes: if a high actual correlation strength between two comparisons was based on many shared genes, the actual correlation coefficient was closer to the expected average than if similar binning averages were driven by different genes within each bin. Since altered gravity-related correlations, especially inter-experiment, showed a low average expected correlation strength, their distribution of differentially expressed genes was fundamentally different to the control groups and based on colocalizing gravity-responsive chromosomal regions that were driven by different genes.

To illustrate these two models (correlation based on pairs of genes vs colocalizing reactive chromosomal regions with different genes), a computational simulation has been performed, comparing the expected and the actual correlation strength for different model parameters. For single correlating genes, the actual vs expected correlation strength was the same, for reactive chromosomal regions with larger differences between the genes, the actual correlation strength was elevated (Supplementary Figure 10).

Supplementary Text 2. Probing the involvement of gene sets as potential underlying explanation.

S2.1 Gene sets as alternative model were not able to describe observed effects

Since the arrangement of mammalian genes in the genome is known to be partly organized by biological function, we analyzed whether the observed patterns could be explained by functional gene sets that were localized closely and activated by one or a few gravitational-force-sensitive pathways. If gene sets were the underlying explanation of the observed effects, those correlations with strong colocalizing GRCR should display high benchmarking values for gene set enrichment analyses. Therefore, a gene set enrichment analysis was performed per data set including all genes of all differential gene sets. Several parameters have been used to characterize the likelihood of gene sets as underlying explanation of the observed patterns (Supplementary Figure 11). Pairs within the top 15 upregulated and the top 15 downregulated Gene Ontology (GO) pathways resulted in a strong overlap of 18/30 shared sets for the two "control experiment 2" comparisons and almost no overlap for the GBFs Jurkat comparisons (GO pathway names are listed in Supplementary Table S3). Real altered gravity intra-experiment comparisons had a given overlap (13/30 for TEXUS-51, 11/30 for 23rd DLR PFC), and inter-experiment showed a reduced overlap (8/30 for hypg, 3/30 for μg). Within the differentially enriched GO gene sets ($p < .05$), the comparisons with the largest overlap have been determined. "Control experiment 1" had the largest overlap (79%) and the inter-experiment comparison for microgravity vs normal gravity had the smallest overlap (4.1%). As a third benchmarking category, the correlation coefficient between enrichment scores for all MSigDB gene sets have been calculated. Again, "control experiment 1" had the strongest correlation and GBFs Jurkat the weakest. Real altered gravity intra-experiment comparisons again were a bit stronger than inter-experiment comparisons. The same analysis was repeated, however, only analyzing those genes that were used for bin average calculation (Supplementary Figure 11b, GO pathway names in Supplementary Table S4). Consistently, comparable results emerged with the strongest gene set effect for "control experiment 1" in all categories and the weakest effect in all categories for the inter-experiment conditions.

Additionally, the correlation coefficient between all benchmarking parameters and the relative difference in correlation strength were calculated, which was around 0 for most and even negative for some categories. Therefore, the results demonstrated that data sets that showed strong shared gravity-responsive chromosomal regions did not display increased gene set enrichment behavior. Thus, functional gene sets might be partially involved, however, were not the determinant underlying explanations of the gravity-responsive chromosomal regions. In contrast, certain functional gene sets might be the strongest underlying mechanisms for the control experiments, which were not gravity related.

Three gene sets emerged as significantly enriched in multiple conditions that were related to transcription, cell adhesion, and G protein activity. These gene sets could be involved in functional mechanisms that could promote transcriptional reactions towards altered gravity. To test whether a general involvement of such genes could be detected, we performed a heatmap analysis of the three GO gene sets GO_TRANSCRIPTION_REGULATOR_ACTIVITY, GO_BIOLOGICAL_ADHESION, and GO_G_PROTEIN_COUPLED_RECEPTOR_SIGNALING_PATHWAY (Supplementary Figure 12). For all gene sets, the fold changes of all genes were plotted for all data sets, independently of p value. The heatmap was clustered on the genes axis to detect any groups of genes that could emerge in parallel. No significant correlation of genes between different gravitational conditions that would be unique to the true altered gravity comparisons sets could be identified.

Supplementary Figure 1. 1D projection of significantly differently expressed genes (false discovery rate-adjusted p value < 0.05) along all chromosomes of selected altered gravity data sets. Corresponding to Figure 2 of the main text.

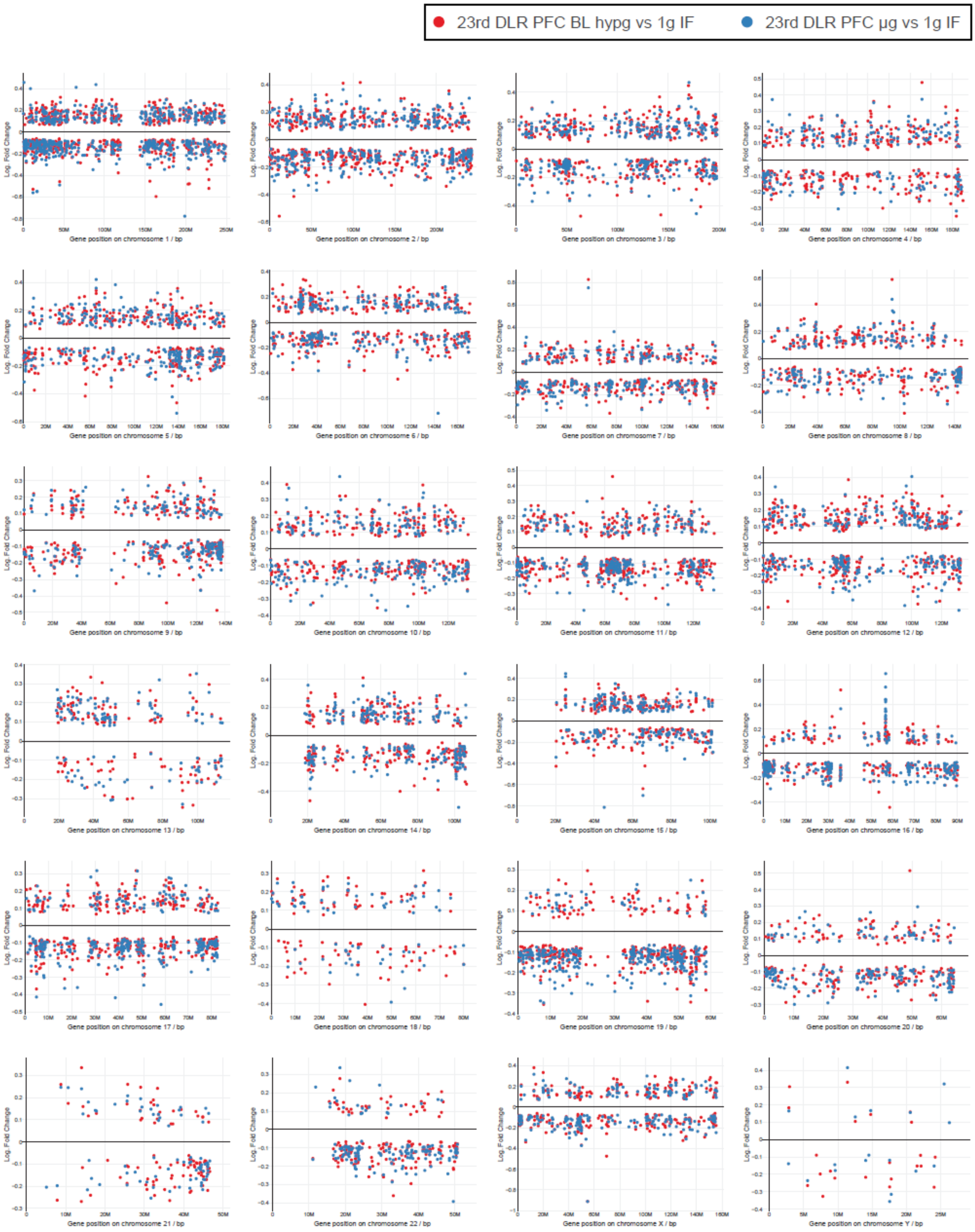

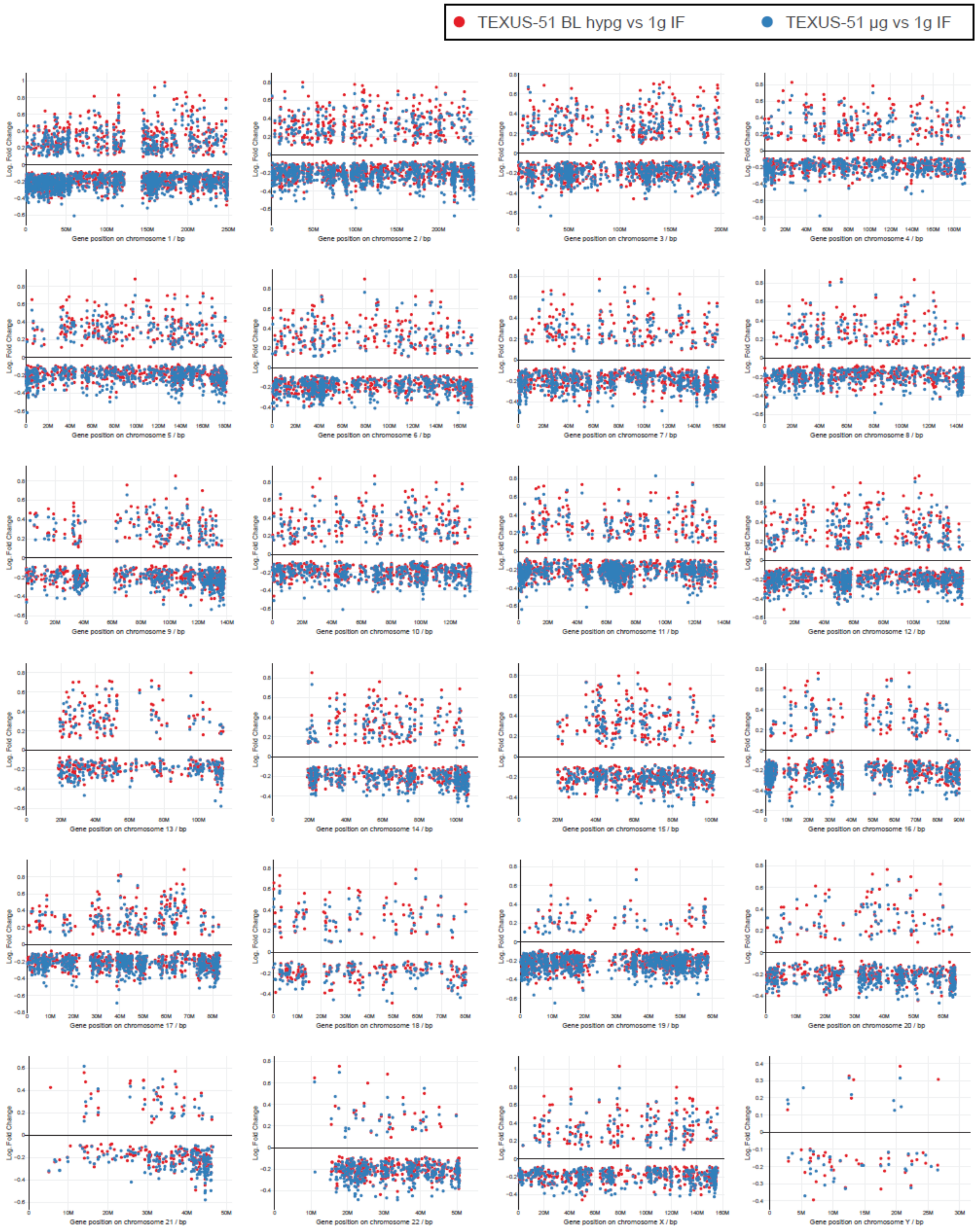

Supplementary Figure 2. Model Hypothesis Testing Extension. Extension of figure 4. Analysis of different potential underlying DNA structures that could be involved in the observed differential gene expression patterns. Each row represents one structural element, the last two rows are combinatory models. The first column "split" describes the structural element-based rule by which the top 2000 differentially expressed genes are separated into two groups. For both subsets, a correlation of regional trends analysis has been performed independently. The following column shows the result of the correlation of regional trends analysis, the Spearman correlation coefficient for the gravity-related intra-experiment comparisons, TEXUS-51 hypergravity vs 1g inflight compared with microgravity vs 1g

inflight and the same comparison for the 23rd DLR PFC experiment. A difference in behavior between split sets a and b indicates an underlying structure in the data represented in those categories. For topology-associated-domains, lamina-associated domains and the mixed models, no categorial split into two categories could be defined that provides enough bins per category to be able to perform this analysis. The column "linear modeling" provides the average R2 predictive strength for the logFC of genes of linear models based on the underlying DNA structures. Fits have been performed on all used data groups separately (columns) and performed independently on datasets including the top 2000/5000/16800 genes. Due to the very rudimentary linear models, no high R2 values are expected. The scale ranges from 1 (perfect predictive strength) over 0 (no predictive strength) and can be arbitrarily lower (prediction worse than random drawing of predictions).

Color coding is from dark red (highest predictive strength for any hypothesis for all experiments, per dataset of top n regulated genes) to white (0.01 or below). Topology-associated domains have a high predictive power for the control experiment 2 and therefore define the upper limit of the relative color scale.

	Split	Cross-correlation: TEXUS-51 (BL hyp-g vs 1g IF; corr µg vs 1g IF), 23rd DLR (BL hyp-g vs 1g IF; corr µg vs 1g IF)	Linear Modeling R² value	TEXUS-51		23rd DLR		GBF Jurkat		Ctrl. Exp. 1		Ctrl. Exp. 2	
				hyp-g 1g IF	µg 1g IF	hyp-g 1g IF	µg 1g IF	hyp-g 1g	sim µg 1g	Sample Gr. 1	Sample Gr. 2	Sample Gr. 1	Sample Gr. 2
**Chromosome lengths**	a) chr1 - chr12, chrX<br>b) chr13 - chr22, chrY	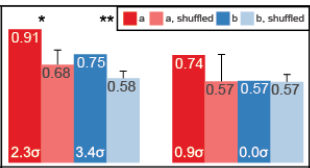	2000 genes	0.07	0.06	0.02	0.02	0.00	0.01	0.03	0.03	0.04	0.01
			5000 genes	0.06	0.06	0.02	0.02	0.01	0.00	0.02	0.02	0.03	0.01
			16800 genes	0.02	0.02	0.01	0.01	0.00	0.00	0.01	0.01	0.01	0.00
**Chromosome ends**	a) outer 1/5 of p/q arm<br>b) rest of chromosome	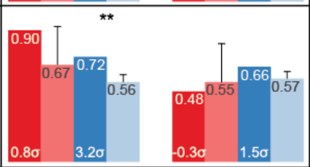	2000 genes	0.07	0.08	0.02	0.01	0.00	0.00	0.00	0.00	0.02	0.01
			5000 genes	0.05	0.05	0.02	0.02	0.00	0.00	0.00	0.00	0.01	0.01
			16800 genes	0.01	0.02	0.01	0.01	0.00	0.00	0.00	0.00	0.01	0.01
**Alu sequence density**	a) >50 Alu seq within 100k bp<br>b) less Alu seq within 100k bp	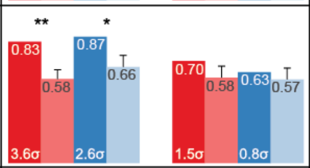	2000 genes	0.01	0.01	0.00	0.00	0.00	0.00	-0.01	-0.01	-0.02	-0.02
			5000 genes	0.00	0.00	0.00	0.00	0.01	0.00	0.00	0.00	0.00	0.00
			16800 genes	-0.09	-0.11	-0.04	-0.03	-0.01	0.00	0.00	0.00	-0.01	-0.01
**Replication domains**	a) early replication<br>b) late replication	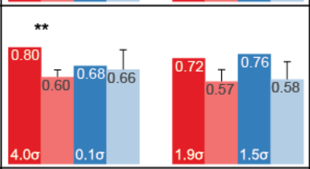	2000 genes	-0.03	-0.01	0.00	-0.01	0.03	0.00	0.00	0.00	-0.03	-0.02
			5000 genes	-0.01	0.00	0.00	-0.01	0.02	0.01	0.00	0.00	-0.01	0.00
			16800 genes	-0.13	-0.15	-0.05	-0.05	-0.01	-0.01	-0.01	0.00	-0.01	-0.01
**Chromosome cytobands**	a) positive bands<br>b) negative bands	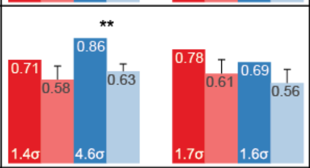	2000 genes	0.10	0.11	0.05	0.04	0.00	0.00	0.04	0.04	0.15	0.18
			5000 genes	0.10	0.10	0.04	0.04	0.01	0.00	0.03	0.02	0.11	0.11
			16800 genes	0.03	0.03	0.02	0.02	0.00	0.00	0.01	0.01	0.05	0.05
**Topology-associated domains**	—	—	2000 genes	0.15	0.16	0.06	0.07	0.02	0.01	0.09	0.07	0.26	0.20
			5000 genes	0.14	0.14	0.04	0.05	0.03	0.02	0.04	0.04	0.18	0.14
			16800 genes	0.04	0.03	0.01	0.01	0.01	0.00	0.02	0.02	0.07	0.06
**Lamina-associated domains**	—	—	2000 genes	0.02	0.02	0.00	0.00	0.00	-0.01	0.01	0.01	0.09	0.06
			5000 genes	0.02	0.01	0.01	0.00	0.00	0.00	0.01	0.00	0.08	0.04
			16800 genes	-0.03	-0.04	-0.01	-0.01	0.00	0.00	0.00	0.00	0.02	0.02
**Chromosome lengths/ends**	—	—	2000 genes	0.14	0.14	0.04	0.04	0.00	0.01	0.04	0.04	0.06	0.04
			5000 genes	0.12	0.12	0.03	0.04	0.00	0.01	0.03	0.03	0.04	0.03
			16800 genes	0.04	0.04	0.02	0.02	0.00	0.00	0.01	0.01	0.02	0.01
**Chrom. lengths /ends/Alu/Rep.**	—	—	2000 genes	0.17	0.16	0.06	0.04	0.03	0.02	0.05	0.05	0.07	0.04
			5000 genes	0.14	0.14	0.05	0.05	0.03	0.02	0.04	0.04	0.05	0.03
			16800 genes	0.05	0.05	0.03	0.03	0.02	0.01	0.02	0.01	0.02	0.02

Supplementary Figure 3. Extension of figure 5. Pinpointing differential gene expression to single chromosomes. (**a-c**) show the percentage of differentially expressed genes, defined as uniquely mapping transcript clusters with uncorrected P<.05 divided by the number of all uniquely mapping transcript clusters per chromosome for (**a**) all differentially expressed genes, (**b**) upregulated genes only, and (**c**) downregulated genes only. (**d-f**) show the elastic net modeling parameter describing the overall log. fold change for the predicted values that is assumed for the entire chromosome in the chromosome length linear model. (**d**) is based on the top 2000 genes, (**e**) is based on the top 5000 differentially expressed genes (top 2500 up, top 2500 down), and (**f**) on all uniquely mapping genes. Chromosomes 18 and 19 are highlighted as reference points with known geometry for Jurkat cells. All conditions are control conditions, not being generated by real altered gravity.

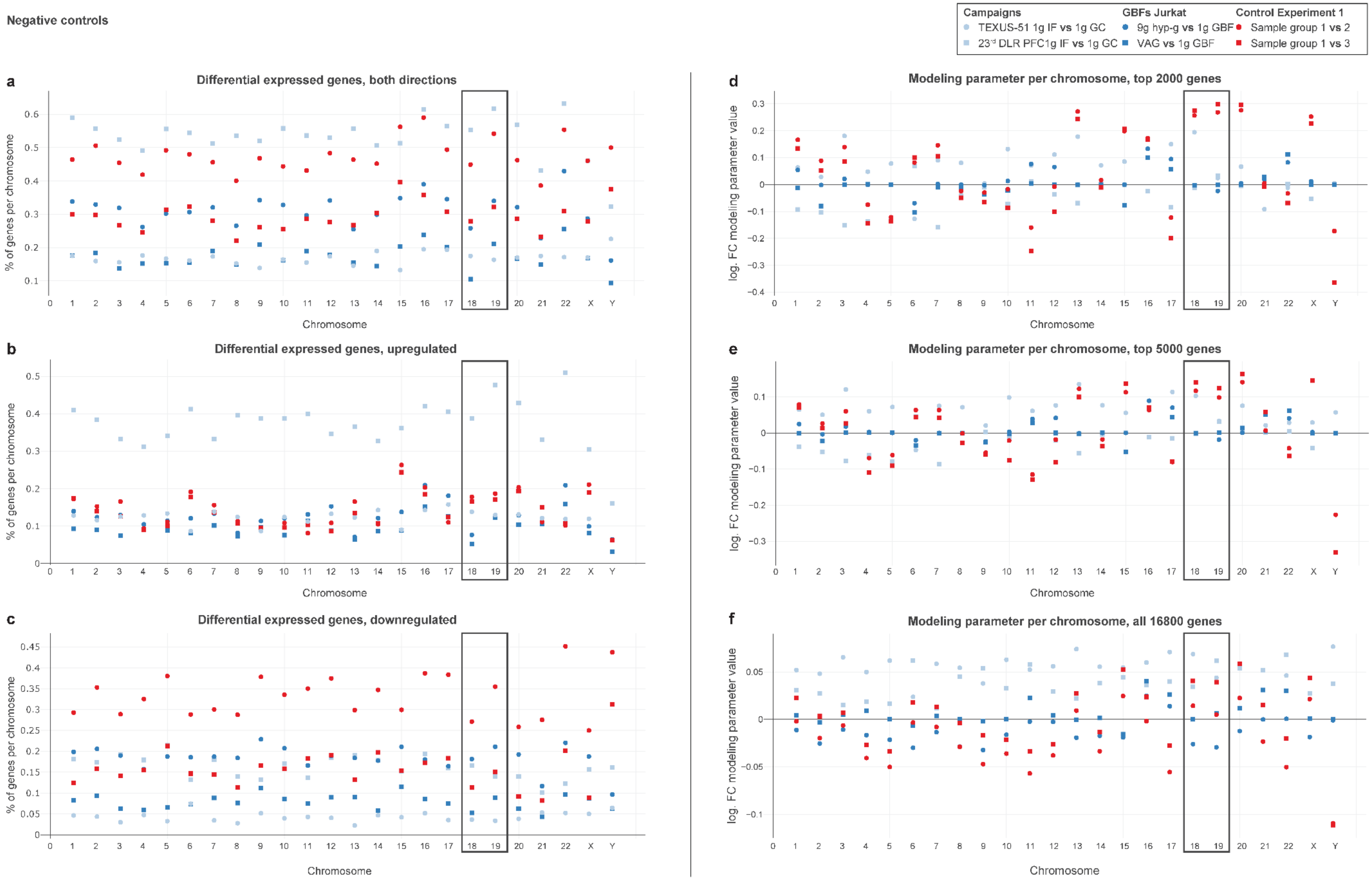

Negative controls
Campaigns
TEXUS-51 1g IF vs 1g GC
23rd DLR PFC1g IF vs 1g GC
GBFs Jurkat
9g hyp-g vs 1g GBF
VAG vs 1g GBF
Control Experiment 1
Sample group 1 vs 2
Sample group 1 vs 3
a
Differential expressed genes, both directions
b
Differential expressed genes, upregulated
c
Differential expressed genes, downregulated
% of genes per chromosome
Chromosome
d
Modeling parameter per chromosome, top 2000 genes
e
Modeling parameter per chromosome, top 5000 genes
f
Modeling parameter per chromosome, all 16800 genes
log. FC modeling parameter value

Supplementary Figure 4. Overlap of gravi-sensitive regions and differential Hi-C bin pairs, full genome. Overlap between gravi-sensitive transcriptional regions from clustering correlation analysis (both 23rd DLR PFC comparisons have a transcriptional binning average for these sites) and significant differential Hi-C bin pairs. Hi-C bin pairs are linked by grey curves. The A/B compartments are highlighted for all three in flight conditions, with 1gIF, hypg and μg in ascending order. The non-randomness of Hi-C bins towards differential transcription clusters over the entire genome has been assessed with a Fisher's exact test as 4.7 x 10-5 for hypg vs 1gIF and as 1.3 x 10-9 for microgravity vs 1gIF.

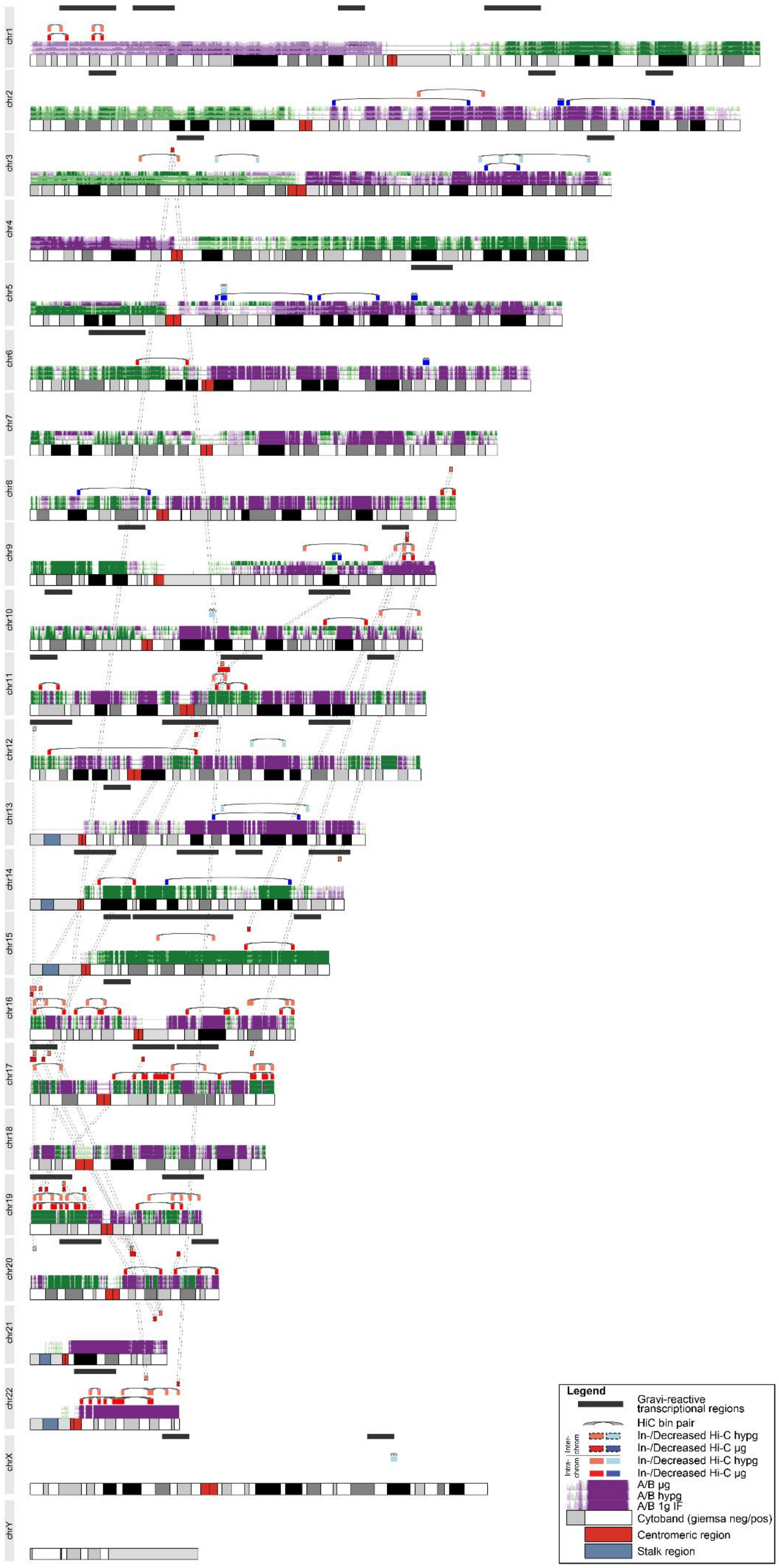
chr1
chr2
chr3
chr4
chr5
chr6
chr7
chr8
chr9
chr10
chr11
chr12
chr13
chr14
chr15
chr16
chr17
chr18
chr19
chr20
chr21
chr22
chrX
chrY
Legend
Gravi-reactive transcriptional regions
HiC bin pair
Inter-chrom
Intra-chrom
In-/Decreased Hi-C hypg
In-/Decreased Hi-C μg
In-/Decreased Hi-C hypg
In-/Decreased Hi-C μg
A/B μg
A/B hypg
A/B 1g IF
Cytoband (giemsa neg/pos)
Centromeric region
Stalk region

Supplementary Figure 5. Correlation of regional trends in differential gene expression (DGE) between intra- and inter-experiment contrasts, expectation vs actual data. (**a**) Chromosomal projection of differential gene expression exemplary shown for DGE comparison TEXUS-51 BL hyp-g vs 1g IF for chromosome 17. The fold changes of transcribed genes between two experimental conditions are mapped against the localization on the linear nucleotide chain, chromosome by chromosome. The genes are non-evenly distributed which is a consequence of the clustered location of genes on human chromosomes and the predefined sampling pattern of the microarray design. The absence of genes at 25M base pairs indicates the centromere of the chromosome. A general broad downregulation trend with spikes of upregulation in certain areas can be observed, potentially indicating gravity-responsive chromosomal regions. (**b**) To be able to analyze distribution and direction of potential gravity-responsive chromosomal regions between comparisons from different experiments, data were filtered, binned and averaged to be able to calculate correlation coefficients between them. The 2000 genes with strongest absolute logarithmic (log) fold change have been considered, filtering out the noisy background. Bins of 10 million base pairs have been defined. Distance between two bins is given as 5 million base pairs, leading to a 50% overlap with neighboring bins. This oversampling prevents missing (gravity-)responsive chromosomal regions by cutting them apart by the arbitrarily chosen bin borders. A binning average has been calculated if a bin contained at least 10 genes (bins 6-14, 16), otherwise no average has been calculated (bins 1-5, 15, 17). To get a measure for the general fold change in a bin, the binning average has been calculated based on the log. fold change weighed by the average expression of genes. Resulting binning average data points have been used for correlation analysis between differential gene sets in the next step. (**c**) Expected Spearman correlation between binned differential gene expression sets from average distribution of fold changes of the genome. The expected correlation coefficient / FDR-adjusted p value has been calculated by bootstrapping 1000 permutations of fold changes between genes within the genome without separating identical genes between comparisons. The lower triangle shows correlation strength (color coded, red: strong correlation, grey: no correlation, blue: strong anticorrelation), the upper triangle shows FDR-adjusted p values of given Spearman correlation (color coded, green: significantly different from zero hypothesis, black: insignificant). The diagonal line is a DGEs comparison's autocorrelation, therefore has correlation coefficient 1 and p value 0. It shows a significant correlation for all intra-experiment comparisons with highest levels for control experiments and the simulated gravity comparisons and is below 0.2 and therefore non-significant for all inter-experiment comparisons. (**d**) Actual Spearman correlation based on performing the same analysis as for c on the observed, non-shuffled distribution. Sets are ordered by clustering of correlation coefficients to all other sets, a dendrogram is given on the right side. Significant intra-experiment correlation can be observed for all experiments, inter-experiment correlation is strongly given between TEXUS-51 and the 23rd DLR PFC, partly between GBFs and TEXUS-51 and weakly (anticorrelation) between TEXUS-51 and control experiment 2.

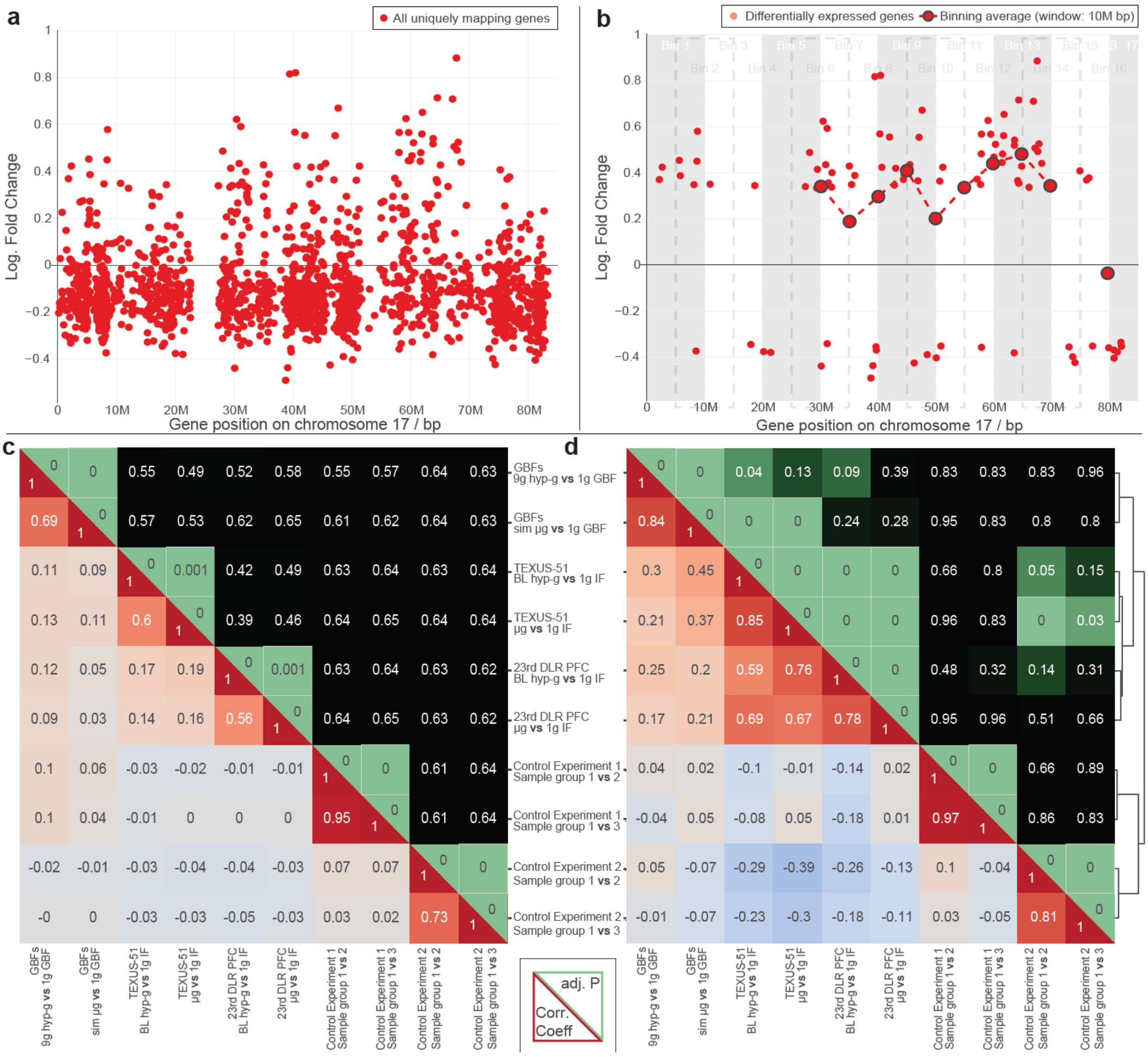
a
All uniquely mapping genes
Log. Fold Change
1
0.8
0.6
0.4
0.2
0
−0.2
−0.4
0 10M 20M 30M 40M 50M 60M 70M 80M
Gene position on chromosome 17 / bp
b
Differentially expressed genes
Binning average (window: 10M bp)
Bin 1 Bin 2 Bin 3 Bin 4 Bin 5 Bin 6 Bin 7 Bin 8 Bin 9 Bin 10 Bin 11 Bin 12 Bin 13 Bin 14 Bin 15 Bin 16 B. 17
Log. Fold Change
1
0.8
0.6
0.4
0.2
0
−0.2
−0.4
0 10M 20M 30M 40M 50M 60M 70M 80M
Gene position on chromosome 17 / bp
c
1 0 | 0 | 0.55 | 0.49 | 0.52 | 0.58 | 0.55 | 0.57 | 0.64 | 0.63
0.69 | 1 0 | 0.57 | 0.53 | 0.62 | 0.65 | 0.61 | 0.62 | 0.64 | 0.63
0.11 | 0.09 | 1 0 | 0.001 | 0.42 | 0.49 | 0.63 | 0.64 | 0.63 | 0.64
0.13 | 0.11 | 0.6 | 1 0 | 0.39 | 0.46 | 0.64 | 0.65 | 0.64 | 0.64
0.12 | 0.05 | 0.17 | 0.19 | 1 0 | 0.001 | 0.63 | 0.64 | 0.63 | 0.62
0.09 | 0.03 | 0.14 | 0.16 | 0.56 | 1 0 | 0.64 | 0.65 | 0.63 | 0.62
0.1 | 0.06 | -0.03 | -0.02 | -0.01 | -0.01 | 1 0 | 0 | 0.61 | 0.64
0.1 | 0.04 | -0.01 | 0 | 0 | 0 | 0.95 | 1 0 | 0.61 | 0.64
-0.02 | -0.01 | -0.03 | -0.04 | -0.04 | -0.03 | 0.07 | 0.07 | 1 0 | 0
-0 | 0 | -0.03 | -0.03 | -0.05 | -0.03 | 0.03 | 0.02 | 0.73 | 1 0
GBFs 9g hyp-g vs 1g GBF
GBFs sim µg vs 1g GBF
TEXUS-51 BL hyp-g vs 1g IF
TEXUS-51 µg vs 1g IF
23rd DLR PFC BL hyp-g vs 1g IF
23rd DLR PFC µg vs 1g IF
Control Experiment 1 Sample group 1 vs 2
Control Experiment 1 Sample group 1 vs 3
Control Experiment 2 Sample group 1 vs 2
Control Experiment 2 Sample group 1 vs 3
adj. P
Corr. Coeff
d
1 0 | 0 | 0.04 | 0.13 | 0.09 | 0.39 | 0.83 | 0.83 | 0.83 | 0.96
0.84 | 1 0 | 0 | 0 | 0.24 | 0.28 | 0.95 | 0.83 | 0.8 | 0.8
0.3 | 0.45 | 1 0 | 0 | 0 | 0 | 0.66 | 0.8 | 0.05 | 0.15
0.21 | 0.37 | 0.85 | 1 0 | 0 | 0 | 0.96 | 0.83 | 0 | 0.03
0.25 | 0.2 | 0.59 | 0.76 | 1 0 | 0 | 0.48 | 0.32 | 0.14 | 0.31
0.17 | 0.21 | 0.69 | 0.67 | 0.78 | 1 0 | 0.95 | 0.96 | 0.51 | 0.66
0.04 | 0.02 | -0.1 | -0.01 | -0.14 | 0.02 | 1 0 | 0 | 0.66 | 0.89
-0.04 | 0.05 | -0.08 | 0.05 | -0.18 | 0.01 | 0.97 | 1 0 | 0.86 | 0.83
0.05 | -0.07 | -0.29 | -0.39 | -0.26 | -0.13 | 0.1 | -0.04 | 1 0 | 0
-0.01 | -0.07 | -0.23 | -0.3 | -0.18 | -0.11 | 0.03 | -0.05 | 0.81 | 1 0

Supplementary Figure 6. Altered analysis parameters. Analysis from figure 2 was performed with altered parameters, keeping all parameters constant except for a not filtering for the top 2000 differentially expressed genes but all 16800 uniquely mapping transcript clusters, b setting the minimum number of transcript clusters per bin to 1, c disabling 2x oversampling through setting the bin distances to 10 million bp, and d setting the bin sizes to 1/10th of the original size, thereby adequately reducing the minimum number of genes per bin to 1. Correlation drops are represented in absolute values (difference of correlation coefficients) and relative values (in multiples of standard deviation).

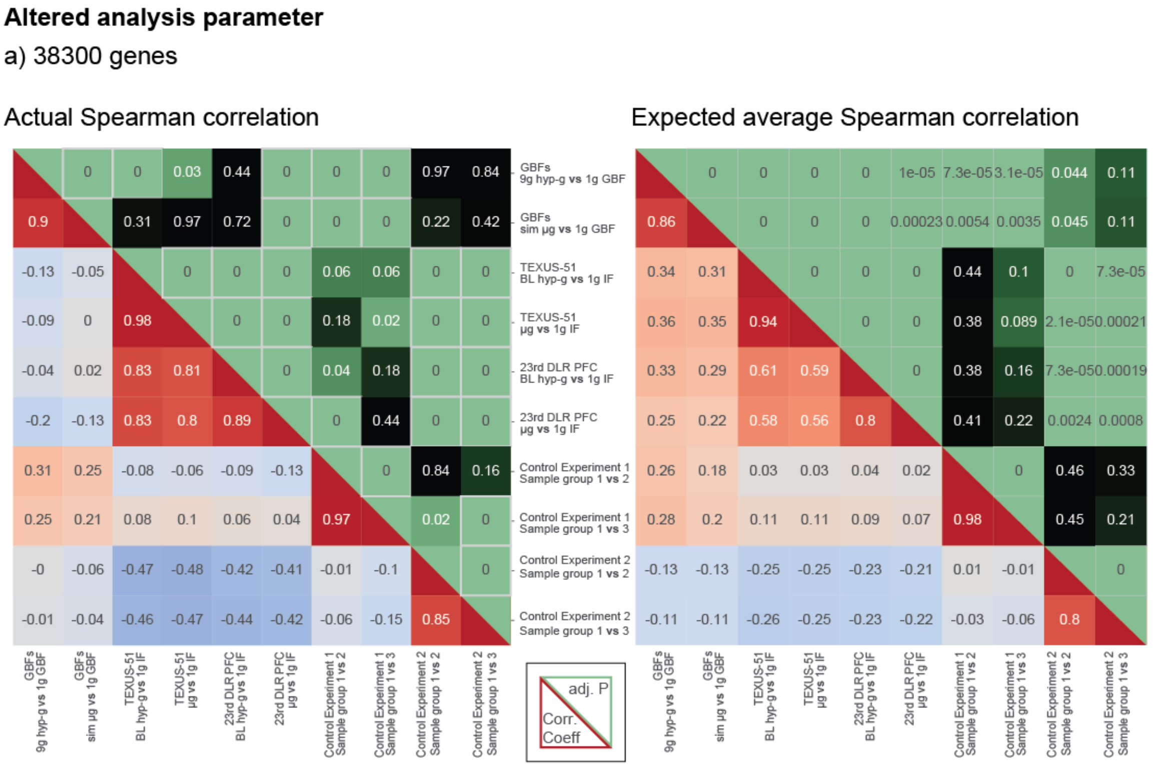

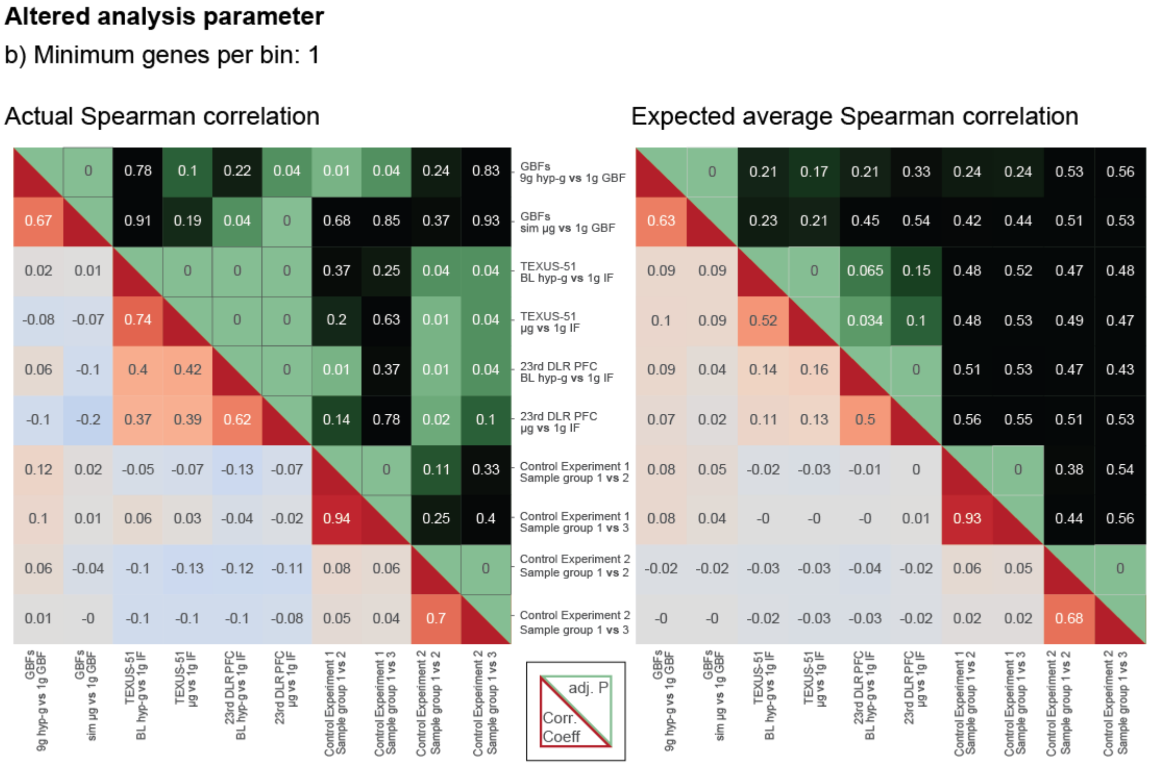

Altered analysis parameter

c) Bin distance 10 mio. bp (no oversampling)

Actual Spearman correlation　　　　Expected average Spearman correlation

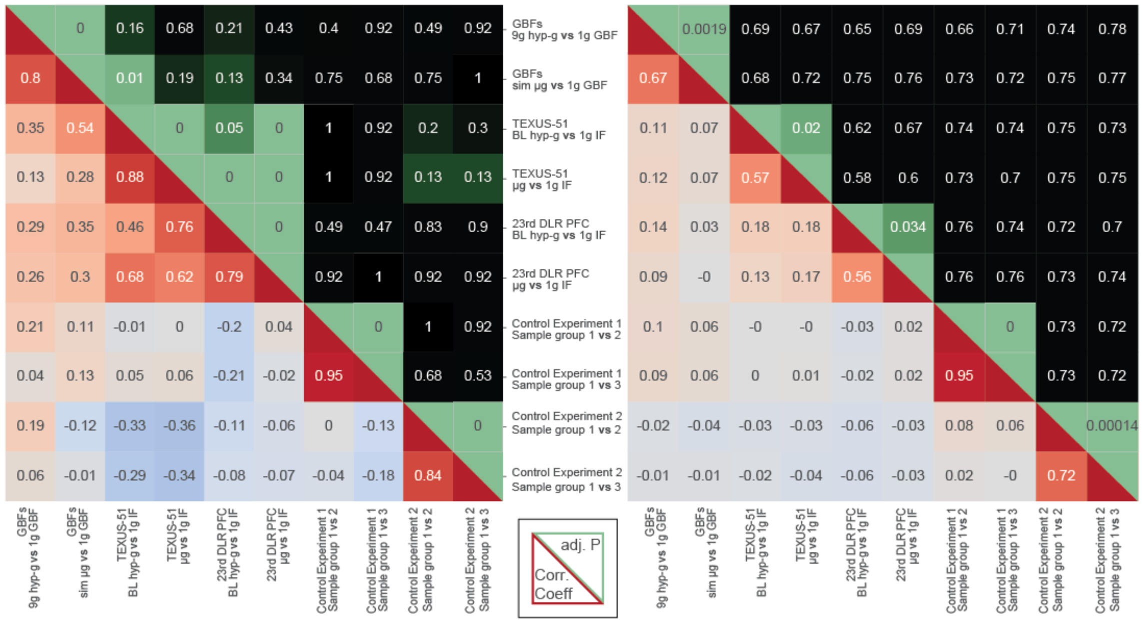

Altered analysis parameter

d) Bin size: 1 mio bp, minimum genes per bin: 1

Actual Spearman correlation　　　　Expected average Spearman correlation

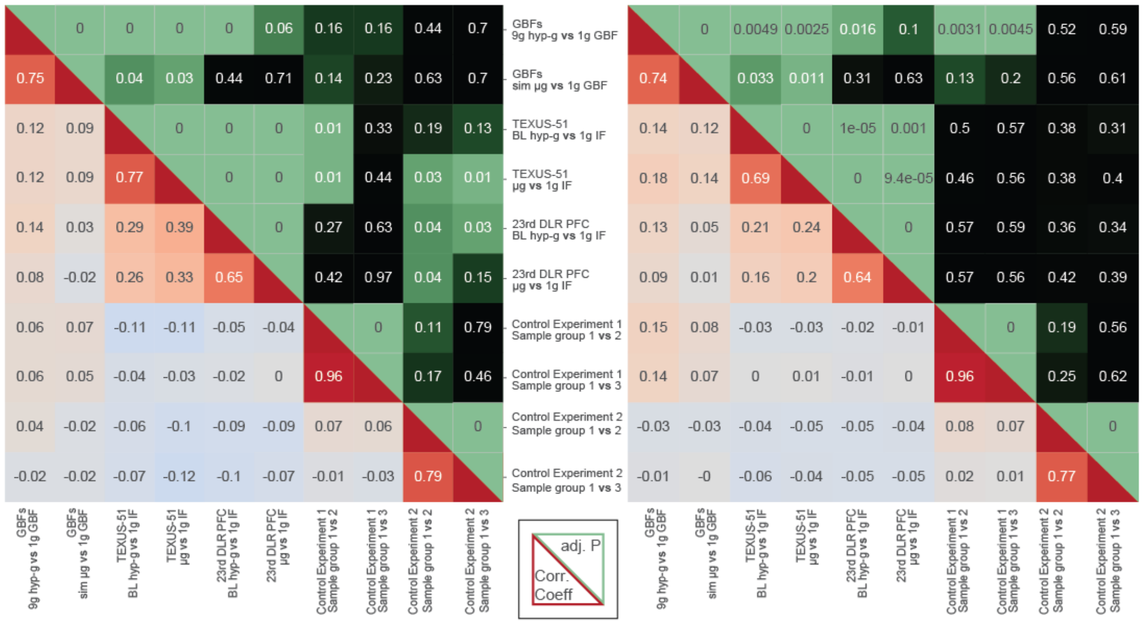

Supplementary Figure 7. Binning Window Sizes. Spearman correlation coefficient analysis with altered bin sizes (and altered minimum genes per bin numbers to compensate for smaller bins). The difference in Spearman correlation coefficients before and after shuffling is visualized for bin sizes from 1 to 20 million bp for different DGE comparisons (altered gravity intra-experiment comparisons, inter-experiment comparisons, control experiment 2 comparisons).

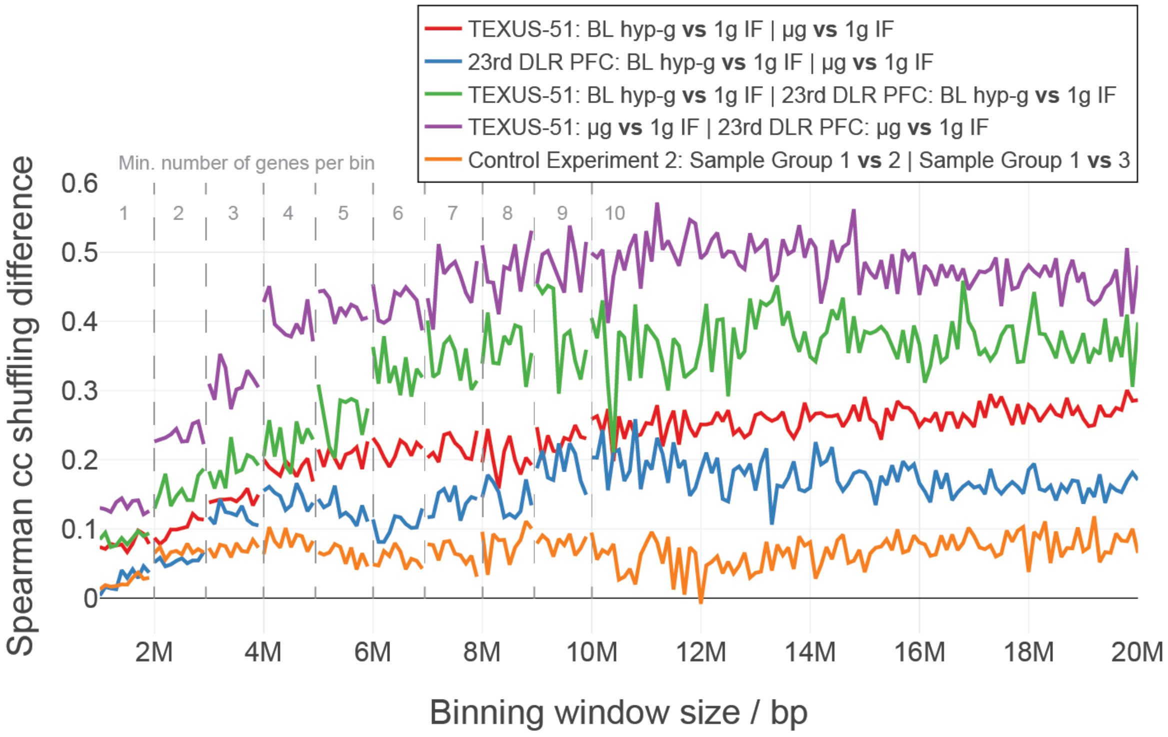

Supplementary Figure 8. Analysis after label permutation. To understand if technical effects or biological effects independent of altered gravity effects as an explanation of the correlation patterns can be ruled out, a label permutation analysis was performed where the 50% of the labels between two compared experiments were exchanged prior to DGE calculation to statistically eliminate the effect of altered gravity. (**a**) Expected average correlation clustermap of all permutations TEXUS-51 BL hyp-g vs 1g IF correlated with TEXU-51 µg vs 1g IF that average out the gravitational effects. (**b**) Actual Clustermap from (a). (**c**) Average correlation coefficients, expected vs actual, of all possible pairs of cross-correlation for each pair of DGEs comparisons. Standard deviations are indicated by error bars. Displayed values are average absolute values, therefore correlation and anticorrelation are treated equally here.

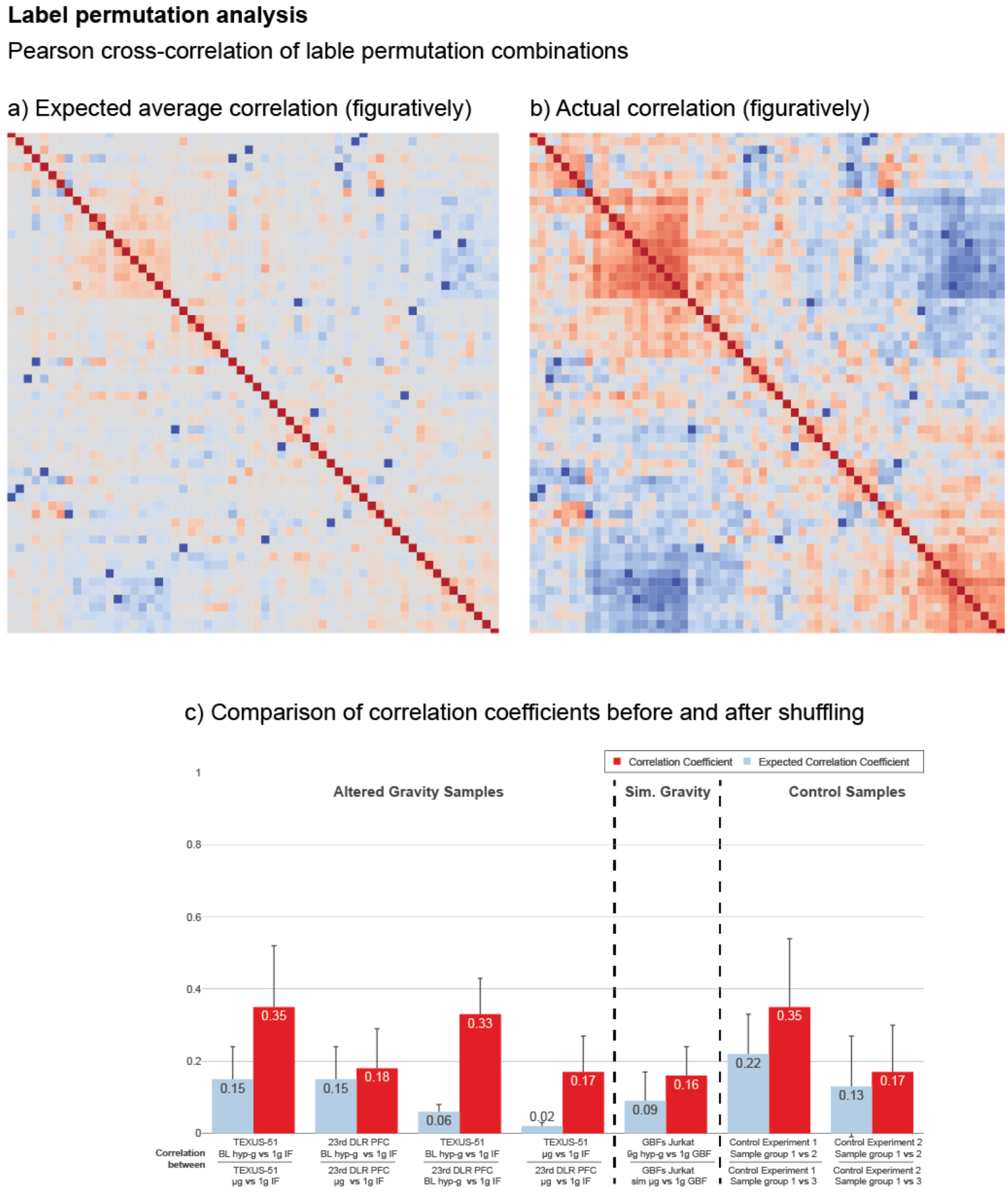

Supplementary Figure 9. Structure of distribution. Fine-structure of differential gene expression between contrasts for one intra-experiment comparison with a strongly increased actual correlation coefficient compared to the expected ((**a**) TEXUS-51 vs 23rd DLR PFC µg vs 1g-IF, Spearman correlation of 0.54 vs 0.17 expected for chromosome 2) and one intra-experiment comparison without ((**b**) GBF 9g hyp-g / sim µg vs 1g, Spearman correlation of 0.69 vs 0.69 expected for chromosome 2). (**c**) LogFC of all genes on the first 50M base pairs of chromosome 2 for two µg comparisons. Both comparisons consist of an uncorrelated background with small fold changes and some genes that show stronger differential expression. If these are present in both comparisons, they are called a local pair. (**d**) Same analysis for the two GBF comparisons. The comparisons show more local pairs than the inter-experiment contrast with smaller differences in logFC. (**e**) The same region after application of the top 2000 differentially expressing genes filter. For the µg comparisons, only 5 local pairs remain, for the GBF comparison, 12 local pairs remain. (**f**) the full chromosome after application of the top 2000 differentially expressing genes filter. For the µg comparison, 20 local pairs are present, for the GBF comparison, 75 local pairs are present.

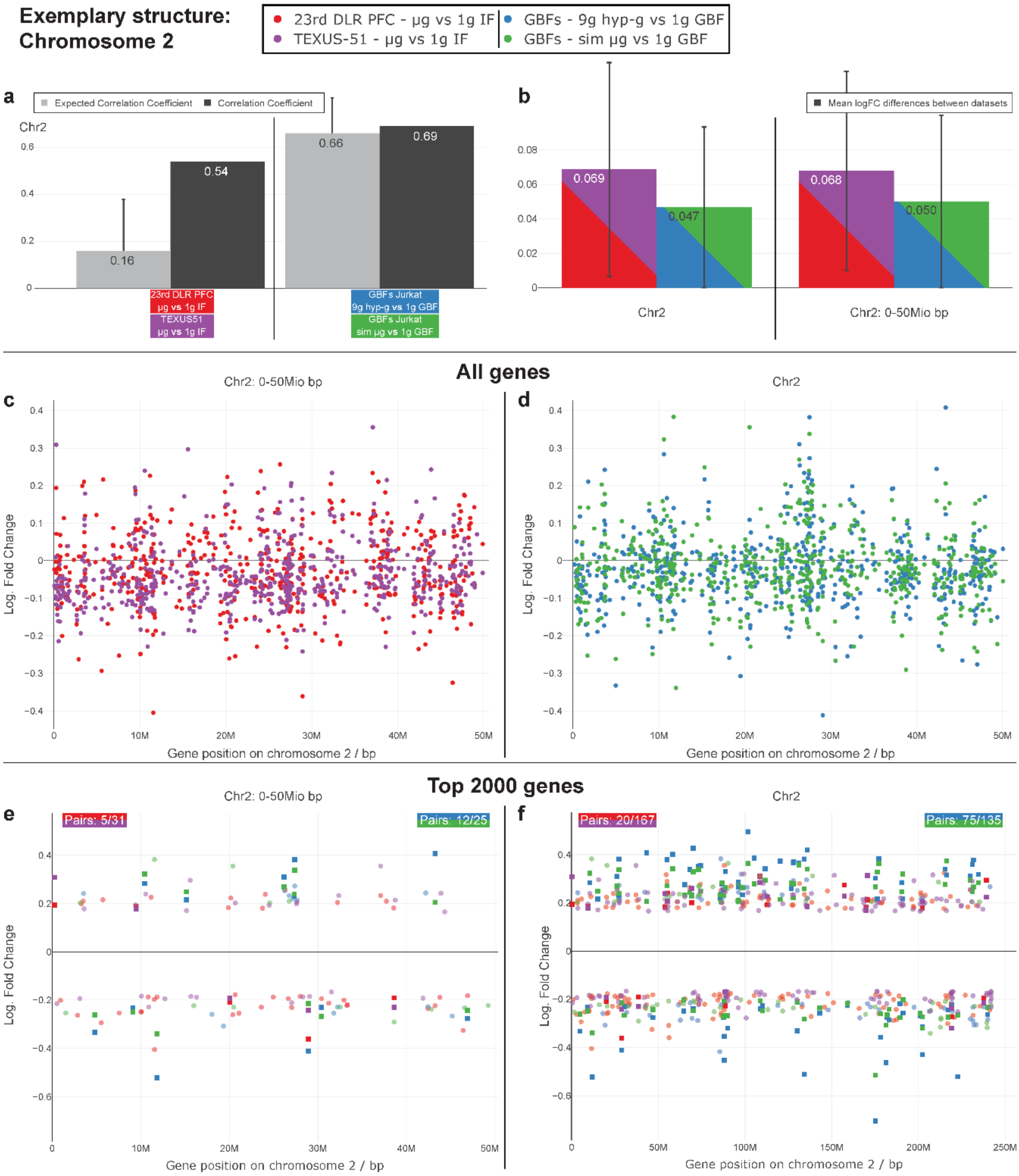

Supplementary Figure 10. Model simulation of distribution. Simulation of two hypothetical different differential gene expression comparisons, based on the genes detectable by the utilized Affymetrix HTA 2.0 microarray. One scenario exclusively depends on single coupled genes, the other on colocalizing regulatory hotspots (GRCRs) with larger differences between genes. Real data sets show a more complex distribution and might be represented by mixture of both cases. (**a**) Scenario 1: Two differential expression sets share a noisy, uncorrelated background, containing the majority of genes. Few genes with high log. fold changes are highly correlated between two sets (indicated by black circles), leading to a comparable binned and averaged signal (large dots) and therefore a high spearman correlation coefficient between those two sets. Schematic figure, not representing real data. (**b**) Spearman correlation between two simulated differential gene sets, following a "strong correlated genes" distribution (comp. Figure 3a. Shown is the actual correlation coefficient and the expected average correlation coefficient resulting from simulated gene sets with different numbers of strong correlated genes. The X axis represents the number of the genes that are highly correlated, the Y axis the resulting correlation coefficient between two sets following the distribution, based on one simulation per number of correlated genes. At 900 highly correlated genes, the correlation coefficient reaches its maximum (exact value depends on simulation settings). The actual coefficient behaves similar to the expected coefficient; therefore, the actual distribution does not behave significantly different than an average permutation. (**c**) Scenario 2: Two different gene sets that share regulatory hotspots of same overall regulation (indicated by black circles). Pairs of genes are not strongly correlated within one geometric region, leading to a higher spread in logFC between the same genes of different sets than for the scenario 1. The emerging binned signal is the same as for the case of strong correlated genes, leading to the same correlation coefficient, rendering this case indistinguishable from the previous one, if only the correlation coefficients are given. Schematic figure, not representing real data. (**d**) Spearman correlation between two simulated differential gene sets, following a "colocalizing regulatory hotspots" distribution. Shown is the correlation coefficient resulting from a simulated gene set. The correlation coefficient between two simulated gene sets is calculated in dependence of the number of geometrical coupling loci. From 100 loci on, the correlation coefficient reaches its maximum (exact values of correlation coefficient depends on simulation settings). The average expected coefficients are significantly lower than the actual coefficients.

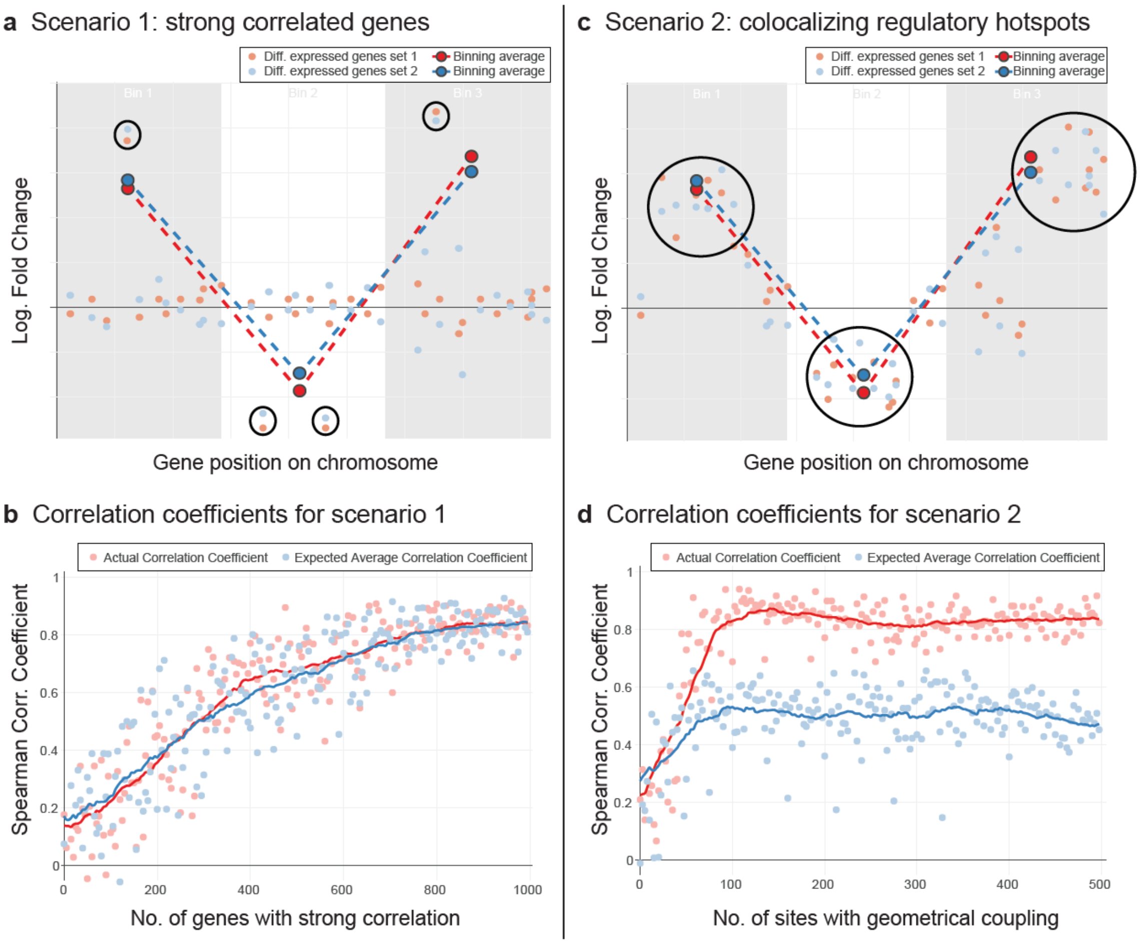

Supplementary Figure 11. Gene set enrichment analysis and comparison within each experiment. To benchmark whether the described correlation effects could be the consequence of gene sets that are functionally enriched in both comparisons for pairs with highly non-average distribution of differential gene expression, a gene set enrichment analysis has been performed and analyzed in four categories: how many pairs are within the 15 top upregulated and the 15 top downregulated GO sets, how many GO sets are differently expressed (with a cutoff value of p 0.05), how many of these GO sets are shared between to comparisons from the same experiment, and how strong the gene set enrichment scores are correlated between two sets from the same experiment. The strongest effect per benchmark category is highlighted in blue, the weakest in red. To statistically test if any of these factors correlates with difference in correlation strength compared to the expected value, the last column contains the Spearman correlation between the tested parameters and the relative drop in correlation in standard deviations. (**a**) Analysis for all genes. No parameter correlates with effect strength. (**b**) Analysis limited to genes that contribute to bin averages within overlapping bins between the two compared columns. No parameter positively correlates with effect strength, some do negatively correlate. Extended tables for a and b with all Top15 up-/downregulated sets can be found in the supplement.

a

All Genes	TEXUS-51 BL hyp-g vs 1g IF	TEXUS-51 µg vs 1g IF	23rd DLR PFC BL hyp-g vs 1g IF	23rd DLR PFC µg vs 1g IF	TEXUS-51 BL hyp-g vs 1g IF	23rd DLR PFC BL hyp-g vs 1g IF	TEXUS-51 µg vs 1g IF	23rd DLR PFC µg vs 1g IF
Pairs within top 30 up-/downregulated pathways	13/30		11/30		8/30		3/30	
Differential GO sets (P<.05)	2734	2704	748	208	2734	748	2704	208
Shared GO Gene Sets (P<.05)	2277 (72%)		132 (16%)		594 (21%)		115 (4.1%)	
CC between shared diff. Gene Set Enrichment Scores	0.57		0.32		0.21		0.1	

All Genes	GBFs Jurkat 9g hyp-g vs 1g GBF	GBFs Jurkat sim µg vs 1g GBF	Control Exp. 1 Sample group 1 vs 2	Control Exp. 1 Sample group 1 vs 3	Control Exp. 2 Sample group 1 vs 2	Control Exp. 2 Sample group 1 vs 3	Correlation with effect strength
Pairs within top 30 up-/downregulated pathways	1/30		11/30		18/30		0.05
Differential GO sets (P<.05)	1210	473	2223	2332	1766	1567	-
Shared GO Gene Sets (P<.05)	209 (14%)		2005 (79%)		1291 (63%)		0.07
CC between shared diff. Gene Set Enrichment Scores	0.07		0.76		0.69		-0.04

b

Shared Genes	TEXUS-51 BL hyp-g vs 1g IF	TEXUS-51 µg vs 1g IF	23rd DLR PFC BL hyp-g vs 1g IF	23rd DLR PFC µg vs 1g IF	TEXUS-51 BL hyp-g vs 1g IF	23rd DLR PFC BL hyp-g vs 1g IF	TEXUS-51 µg vs 1g IF	23rd DLR PFC µg vs 1g IF
Shared genes for CC	694		434		216		185	
Pairs within top 30 up-/downregulated pathways	4		1		0		0	
Differential GO sets (P<.05)	134	46	27	7	10	4	0	5
Shared GO Gene Sets (P<.05)	36 (25%)		1 (3%)		1 (8%)		0 (0%)	
CC between shared diff. Gene Set Enrichment Scores	0.3		0.16		-0.07		0	

Shared Genes	GBFs Jurkat 9g hyp-g vs 1g GB	GBFs Jurkat sim µg vs 1g GBF	Control Exp. 1 Sample group 1 vs 2	Control Exp. 1 group 1 vs 3	Control Exp. 2 group 1 vs 2	Control Exp. 2 group 1 vs 3	Correlation with effect strength
Shared genes for CC	734		968		688		-
Pairs within top 30 up-/downregulated pathways	1		13		4		-0.42
Differential GO sets (P<.05)	17	19	212	192	40	49	-0.25
Shared GO Gene Sets (P<.05)	1 (3%)		174 (76%)		30 (51%)		-0.13
CC between shared diff. Gene Set Enrichment Scores	0.01		0.79		0.62		-0.46

Supplementary Figure 12 Clustered heatmap analysis of the three GO gene sets (a) GO_TRANSCRIPTION_REGULATOR_ACTIVITY, (b) GO_BIOLOGICAL_ADHESION, and (c) GO_G_PROTEIN_COUPLED_RECEPTOR_SIGNALING_PATHWAY. For each pathway, all genes that could be detected on the microarrays was plotted, independently of the corresponding p value. Data was clustered on the genes axis to detect any groups of genes that emerged in parallel exclusively in the altered gravity data sets. Fold changes were plotted color-coded between -0.3 (and below) and 0.3 (and above) with 0 as no color.

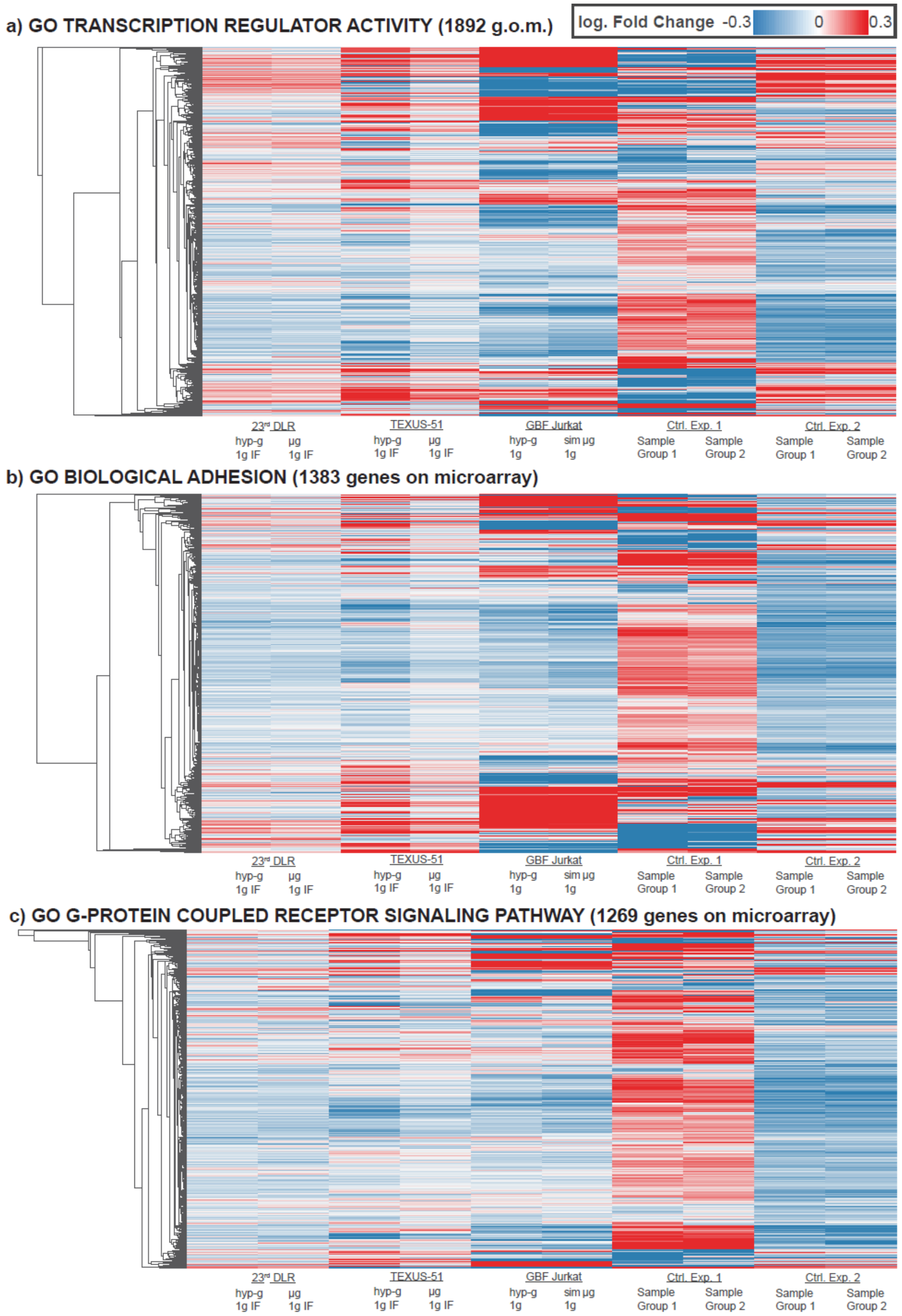

Supplementary Table 1 Significant Hi-C Bin Pairs. In separate excel table.

Supplementary Table 2 Key statistical values of all datasets that have been utilized in this study. Maximum and minimum log2 fold changes are displayed for both positive and negative values. Additionally, the mean absolute fold changes and p values were calculated for each dataset. The maximum and minimum value per category is displayed in green and red.

	**TEXUS-51 BL hyp-g vs 1g IF**	**TEXUS-51 µg vs 1g IF**	**23rd DLR PFC BL hyp-g vs 1g IF**	**23rd DLR PFC µg vs 1g IF**	**GBFs Jurkat 9g hyp-g vs 1g GBF**	**GBFs Jurkat sim µg vs 1g GBF**	**Control Exp. 1 Sample group 1 vs 2**	**Control Exp. 1 Sample group 1 vs 3**	**Ctrl. Exp. 2 Sample group 1 vs 2**	**Ctrl. Exp. 2 Sample group 1 vs 3**
**max pos. log2 FC**	0.504	0.5614	0.827	0.753	0.606	0.670	2.373	2.226	1.269	1.670
**min pos. log2 FC**	0.163	0.170	0.181	0.181	0.219	0.207	0.245	0.232	0.215	0.215
**max neg. log2 FC**	-0.253	-0.382	-0.912	-0.913	-1.105	-0.831	-1.915	-1.770	-1.729	-2.032
**min neg. log2 FC**	-0.163	-0.170	-0.181	-0.181	-0.219	-0.207	-0.245	-0.232	-0.216	-0.215
**mean abs. FC**	0.222	0.212	0.231	0.232	0.299	0.271	0.432	0.406	0.362	0.353
**mean p value**	0.0005	0.0008	0.0223	0.0381	0.0007	0.0243	0.00001	0.0001	0.0022	0.0049

Supplementary Table 3. Shared GSEA pathways. In separate excel table.

Supplementary Table 4. Shared GSEA pathways of overlapping genes. In separate excel table.
